# A serine-conjugated butyrate prodrug with high oral bioavailability suppresses autoimmune arthritis and neuroinflammation in mice

**DOI:** 10.1038/s41551-024-01190-x

**Published:** 2024-04-01

**Authors:** Shijie Cao, Erica Budina, Michal M. Raczy, Ani Solanki, Mindy Nguyen, Taryn N. Beckman, Joseph W. Reda, Kevin Hultgren, Phillip S. Ang, Anna J. Slezak, Lauren A. Hesser, Aaron T. Alpar, Kirsten C. Refvik, Lucas S. Shores, Ishita Pillai, Rachel P. Wallace, Arjun Dhar, Elyse A. Watkins, Jeffrey A. Hubbell

**Affiliations:** 1 Pritzker School of Molecular Engineering, University of Chicago, Chicago, IL USA; 2 Department of Pharmaceutics, School of Pharmacy, University of Washington, Seattle, WA USA; 3 Animal Resource Center, University of Chicago, Chicago, IL USA; 4 Committee on Immunology, University of Chicago, Chicago, IL USA; 5 Committee on Cancer Biology, University of Chicago, Chicago, IL USA

**Keywords:** Molecular engineering, Drug delivery, Autoimmunity, Preclinical research

## Abstract

Butyrate—a metabolite produced by commensal bacteria—has been extensively studied for its immunomodulatory effects on immune cells, including regulatory T cells, macrophages and dendritic cells. However, the development of butyrate as a drug has been hindered by butyrate’s poor oral bioavailability, owing to its rapid metabolism in the gut, its low potency (hence, necessitating high dosing), and its foul smell and taste. Here we report that the oral bioavailability of butyrate can be increased by esterifying it to serine, an amino acid transporter that aids the escape of the resulting odourless and tasteless prodrug (*O*-butyryl-l-serine, which we named SerBut) from the gut, enhancing its systemic uptake. In mice with collagen-antibody-induced arthritis (a model of rheumatoid arthritis) and with experimental autoimmune encephalomyelitis (a model of multiple sclerosis), we show that SerBut substantially ameliorated disease severity, modulated key immune cell populations systemically and in disease-associated tissues, and reduced inflammatory responses without compromising the global immune response to vaccination. SerBut may become a promising therapeutic for autoimmune and inflammatory diseases.

## Abstract (web summary)

The esterification of butyrate to serine makes for an odourless and tasteless oral prodrug that ameliorated disease severity and reduced inflammatory responses in mouse models of rheumatoid arthritis and multiple sclerosis.

## Main

The gut microbiome has been associated with numerous diseases, with one of the key mechanisms involving immune regulation through the production of microbial metabolites^1–4^. Among these metabolites, short-chain fatty acids (SCFAs), such as butyrate, have gained extensive attention owing to their anti-inflammatory and immunomodulatory properties^5–7^. Butyrate is derived from the microbial fermentation of dietary fibre in the colon and serves as a primary energy source for colonocytes and maintains intestinal homeostasis^5,8^. It is essential for protecting intestinal barrier function by facilitating tight junction assembly^9,10^. As an epigenetic modulator, butyrate is a histone deacetylase (HDAC) inhibitor and can thus alter chromatin structures and regulate gene expression^11–13^. Through HDAC inhibition, butyrate has been shown to upregulate forkhead box P3 (Foxp3)—a transcription factor involved in the development and function of regulatory T (T_reg_) cells—as well as suppress NFκB activation, inhibit the production of interferon-γ (IFNγ) and upregulate PPARγ^14–17^. In addition to its broad anti-inflammatory activity, butyrate affects immune cell migration, adhesion, cytokine expression, proliferation, activation and apoptosis^18^. Apart from HDAC inhibition, butyrate can also exert anti-inflammatory effects on immune cells, such as dendritic cells (DCs) and T_reg_ cells, via signalling through specific G-protein-coupled receptors: GPR41, GPR43 and GPR109A^19–22^. Collectively, these properties of butyrate hold promising potential for the development of therapeutic strategies, particularly in the treatment of immunological disorders, including autoimmune diseases.

Autoimmune diseases, which affect nearly 5% of the global population, have increased in prevalence over the past few decades^23^. These disorders arise when the immune system mistakenly attacks the body’s own cells and tissues, resulting in chronic inflammation and tissue damage. The onset of autoimmune diseases may be influenced by a combination of genetic and environmental factors. Recent studies have underscored the pivotal role of the gut microbiome in modulating immune responses and influencing the development and progression of autoimmune diseases^3^. For instance, dysregulation of the gut microbiome, or dysbiosis, has been implicated in the pathogenesis of rheumatoid arthritis and multiple sclerosis^24–27^. Current therapeutics for autoimmune diseases, such as immunosuppressive agents, can provide symptom relief but often do not address underlying causes of these complex disorders. There is accumulating evidence that microbial metabolites, such as SCFAs, can impact the immune system and contribute to the development or regulation of autoimmune diseases^28^. Consequently, these findings highlight the potential of using microbial metabolites as therapeutic agents to treat autoimmune diseases by targeting the underlying mechanisms and modulating the immune response.

Despite this promise, oral administration of sodium butyrate (NaBut) has faced challenges owing to its foul, persistent odour and taste, even when administered with enteric coatings or encapsulation. Moreover, butyrate is not absorbed in the gut regions where it could exert therapeutic effects and is rapidly metabolized in the gut as an energy source, limiting its pharmacological impact^29^. Alternative routes of butyrate administration, such as intrarectal delivery or continuous intravenous infusion, are often deemed unfeasible for patients with chronic disorders^30–33^. Therefore, there is a need for innovative delivery methods for butyrate, including prodrugs that can enhance its systemic bioavailability.

To overcome these limitations, we sought to develop a prodrug strategy that enables butyrate to bypass metabolism in the gut, enter the bloodstream and exert its therapeutic effects systemically after liberation. In this study, we designed an l-serine (l-Ser) conjugate of butyrate (*O*-butyryl*-*l-serine, or here SerBut) that exploits the gut transport mechanisms of amino acids. In addition, the conjugation effectively masked the odour and taste of free butyrate, which is important for facilitating patient compliance for potential clinical applications. We found that SerBut not only enhanced systemic bioavailability but also facilitated its crossing of the blood–brain barrier (BBB), thereby enabling access to the central nervous system (CNS). In a mouse model of collagen-antibody-induced arthritis (CAIA), SerBut treatment showed a substantial reduction in disease progression that was associated with a systemic increase in T_reg_ cells and an increase in the ratio of immunoregulatory M2 macrophages to pro-inflammatory M1 macrophages. In an experimental autoimmune encephalomyelitis (EAE) model of multiple sclerosis, prophylactic, but not therapeutic, administration of SerBut substantially prevented disease development and severity, decreased immune cell infiltration in the spinal cord and upregulated inhibitory markers such as programmed cell death protein 1 (PD-1) and cytotoxic T-lymphocyte associated protein 4 (CTLA-4) on CD4^+^ T cells, increased T_reg_ cells and downregulated activation markers on a variety of myeloid cells in the spinal cord-draining lymph nodes (SC-dLNs). Even though SerBut suppressed immunopathological responses in two autoimmune disease models, in a non-inflammatory setting, SerBut treatment did not reduce vaccinal immune responses at either the humoral or cellular levels.

## Results

### Conjugation of l-Ser to butyrate maintained its biological activity while enhancing oral bioavailability

**Fig. 1:**
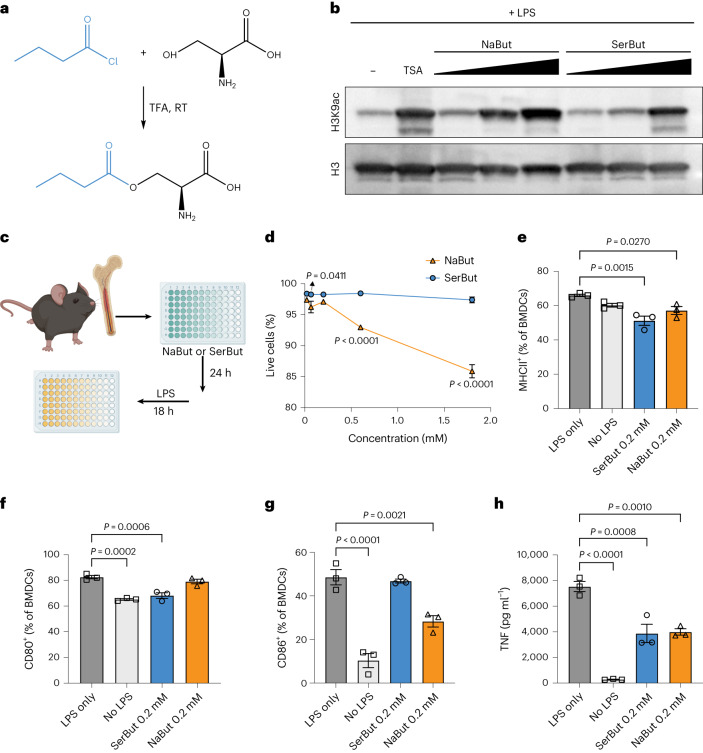
Conjugation of l-Ser to butyrate maintained its biological activity. **a**, Chemical synthesis of serine conjugate with butyrate (SerBut). TFA, trifluoroacetic acid. RT, room temperature. **b**, Whole cell lysates of Raw 264.7 macrophages stimulated with the indicated concentrations of NaBut, SerBut or trichostatin A (TSA) as well as 100 ng ml^−1^ LPS for 18 h were probed for histone acetylation activity via western blot. **c**, Experimental schema on BMDCs incubated with NaBut or SerBut at a series of concentrations for 24 h, followed by LPS stimulation for 18 h. **d**, Percentage of live BMDCs after treatment. **e**–**g**, Percentage of MHC class II^+^ (**e**), CD80^+^ (**f**) or CD86^+^ (**g**) BMDCs analysed by flow cytometry. **h**, TNF concentration in the cell culture supernatant of BMDCs. *n* = 3; data are representative of two independent experiments. Data represent mean ± s.e.m. Statistical analyses were performed using one-way ANOVA with Dunnett’s test. *P* values less than 0.05 are shown. Panel **c** created with BioRender.com. Source data

We synthesized SerBut by conjugating l-Ser to butyryl chloride using trifluoroacetic acid at room temperature, achieving a 79% yield (Fig. 1a and Supplementary Fig. 1). The conjugation effectively masked the unpleasant odour associated with free NaBut or butyrate acid. To assess the HDAC inhibitory activity of SerBut, a property well recognized in butyrate^19,34^, we performed an in vitro histone acetylation assay on the Raw 264.7 macrophage cell line. Our results indicate that SerBut retains HDAC inhibitory activity; however, HDAC inhibition is somewhat reduced compared with NaBut at equivalent concentrations (Fig. 1b). We propose that during the 18 h incubation period, SerBut is subject to enzymatic hydrolysis, sequentially releasing butyrate that may, in turn, exert more substantial HDAC inhibition. This is consistent with our design that SerBut escapes the gut and is subsequently hydrolysed.

Butyrate is known to regulate myeloid cells, including the inhibition of DC maturation in response to pro-inflammatory stimuli^35^. We used bone marrow-derived dendritic cells (BMDCs) to assess the biological effects of SerBut compared with free NaBut (Fig. 1c). BMDCs were first incubated with butyrate formulations for 24 h and then stimulated with lipopolysaccharide (LPS) for 18 h. We found that NaBut showed cytotoxicity to BMDCs at butyrate concentrations above 0.5 mM, while SerBut was well tolerated up to 1.8 mM (Fig. 1d). Flow cytometry was used to compare the expression of the BMDC surface markers major histocompatibility complex (MHC) class II, CD80 and CD86. At the same concentration of 0.2 mM NaBut, SerBut showed similar suppression levels of MHC class II and CD80 (Fig. 1e,f). Although SerBut did not suppress CD86 expression as effectively as NaBut at 0.2 mM (Fig. 1g), we observed dose-dependent suppression of CD86 with SerBut at higher concentrations (Supplementary Fig. 2b). Similarly, SerBut suppressed the secretion of tumour necrosis factor (TNF) from BMDCs (Fig. 1h and Supplementary Fig. 2d). As demonstrated by the HDAC inhibition assay in Raw 264.7 macrophages and the suppression of LPS-stimulated BMDC activation, SerBut retains the biological activity of butyrate.

**Fig. 2:**
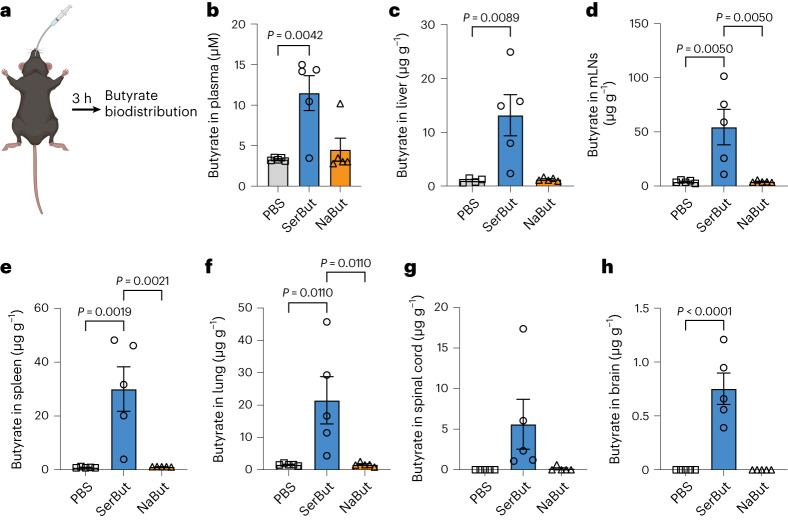
Butyrate biodistribution after SerBut or NaBut oral administration to C57BL/6 mice. **a**, Blood was collected by cheek bleeding at 3 h post oral gavage. Mice were killed and perfused at 3 h post oral gavage, and organs were collected for butyrate quantification. **b**–**h**, The amount of butyrate was detected and quantified in the plasma (**b**), liver (**c**), mLNs (**d**), spleen (**e**), lung (**f**), spinal cord (**g**) and brain (**h**). Butyrate was derivatized with 3-nitrophenylhydrazine and quantified by LC-MS/MS. *n* = 5 mice per group. Experiments were repeated twice; data are representative of two independent experiments. Data represent mean ± s.e.m. Statistical analyses were performed using one-way ANOVA with Dunnett’s test. *P* values less than 0.05 are shown. Panel **a** created with BioRender.com. Source data

The primary site for amino acid absorption is the small intestine, where amino acid transporters are present in intestinal epithelial cells^36^. We conducted a biodistribution study to determine whether conjugating l-Ser to butyrate enhances oral butyrate absorption and bioavailability. We measured free butyrate levels in plasma and several major organs after oral gavage of SerBut and compared them with NaBut (Fig. 2). We observed that SerBut significantly elevated plasma butyrate levels as early as 30 min following oral administration compared with NaBut (Supplementary Fig. 4). At 3 h post-administration, plasma butyrate levels remained higher in the SerBut-treated group than in the NaBut-treated group (Fig. 2b). In the liver, where orally administered drugs enter directly through the hepatic portal circulation, we observed elevated butyrate levels in mice treated with SerBut but not NaBut (Fig. 2c). In the secondary lymphoid organs, we observed elevated butyrate in the mesenteric lymph nodes (mLNs) and spleen (Fig. 2d,e). Butyrate levels were also elevated in lungs at 3 h post-administration of SerBut (Fig. 2f).

l-Ser is an amino acid known to cross the BBB via the sodium-dependent system A and the sodium-independent alanine, serine and cysteine transport system^37,38^. A previous study showed that utilizing the l-type amino acid transporter 1 enhances the efficient delivery of small prodrug across the BBB^39^. Therefore, we investigated whether l-Ser conjugation could assist butyrate in entering the CNS, including the spinal cord and brain. Our findings revealed that SerBut significantly increased butyrate levels in the spinal cord, reaching approximately 43% of the levels found in the liver when normalized for tissue weight, and in the brain, to about 6%. By contrast, no butyrate was detected in these CNS regions following NaBut administration (Fig. 2g,h).

### SerBut suppresses CAIA in mice

**Fig. 3:**
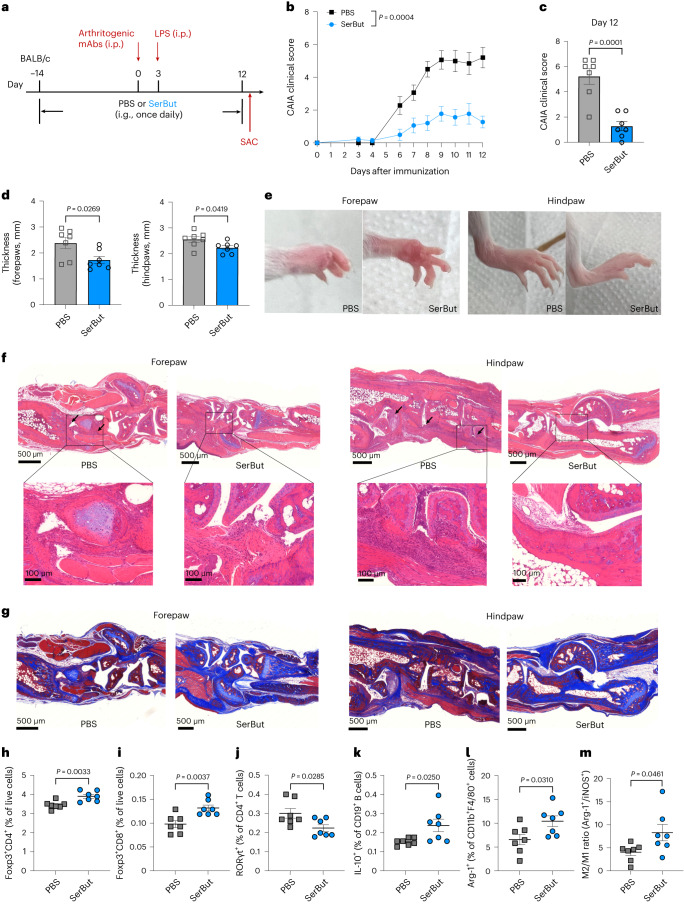
SerBut suppresses arthritis development. **a**, Experimental schema of the CAIA model. Mice were orally gavaged with PBS or SerBut (25 mg) once daily starting on day −14. CAIA was induced by passive immunization with anti-collagen antibody cocktails on day 0, followed by i.p. injection of LPS. i.g., intragastric administration; SAC, mice sacrificed. **b**, Arthritis scores in mice measured daily from day 3 after immunization. **c**, Arthritis scores from PBS- or SerBut-treated mice on day 12. **d**, The thickness of forepaws or hindpaws measured on day 12 from mice treated with PBS or SerBut. **e**, Representative photos of paws after treatment. **f**, Representative images of mouse joints from paws stained with haematoxylin and eosin on day 12 in each treatment group. The arrows indicate immune cell infiltration. **g**, Representative images of mouse joints from paws stained with Masson’s trichrome on day 12. The blue colour represents collagen staining. **h**,**i**, Percentage of Foxp3^+^ regulatory CD4^+^ T cells (**h**) or Foxp3^+^ regulatory CD8^+^ T cells (**i**) of live cells in the spleen measured by flow cytometry. **j**–**m**, Percentage of RORγt^+^ of CD4^+^ T cells (**j**) and IL-10^+^ of CD19^+^ B cells (**k**), as well as Arg-1^+^ of CD11b^+^F4/80^+^ macrophages (**l**) and M2/M1 macrophage ratio (**m**) in the hock-draining LNs. *n* = 7 mice per group. Experiments were repeated twice, and data are representative of two independent experiments. Data represent mean ± s.e.m. Statistical analyses were performed using Student’s *t*-test. Source data

The composition of gut microbiota has been shown to affect the development of rheumatoid arthritis^24,25,40^. Previous research has shown that butyrate can improve rheumatoid arthritis symptoms by targeting key immune cells such as osteoclasts and T cells^41^. Furthermore, studies have demonstrated that butyrate supplementation effectively reduces arthritis severity in animal models by modulation of regulatory B cells^42^. Given SerBut’s enhanced accumulation in crucial immune tissues following oral administration, we sought to evaluate its efficacy in the CAIA mouse model (Fig. 3a). This model is induced by passive immunization with a cocktail of anti-collagen antibodies, followed by LPS, which triggers a cascade of innate immune cell infiltration to the joints, leading to inflammation and swelling within 1 week of immunization.

Mice were pretreated with SerBut or phosphate-buffered saline (PBS) once daily by oral gavage, beginning 2 weeks before the induction of arthritis. We observed that SerBut treatment significantly suppressed the development of arthritis in treated mice. By contrast, PBS-treated mice showed severe inflammation in the fore- and hindpaws, demonstrated by their increasing clinical scores over time and increasing paw thickness (Fig. 3b–e). Histological analysis revealed that oral administration of SerBut effectively suppressed inflammatory responses in the paws and reduced joint pathology compared with PBS-treated mice (Fig. 3f,g). In the CAIA model, the anti-collagen antibodies that bind to joint cartilage activate complement proteins, leading to the recruitment of immune cells such as macrophages and T cells to the affected joints^43,44^. This immune cell infiltration contributes to joint inflammation, tissue damage and the clinical manifestations of arthritis. In our study, we observed that SerBut treatment effectively reduced immune cell infiltration into the joints (Fig. 3f and Supplementary Fig. 5). In addition, anti-collagen antibodies specifically target and degrade collagens in the joints, causing cartilage integrity and joint function loss, ultimately leading to the onset of arthritis symptoms^45^. We found that SerBut treatment also prevented collagen loss in the joints (Fig. 3g and Supplementary Fig. 6), suggesting that SerBut may have therapeutic potential in mitigating joint damage and preserving collagen content in the autoimmune arthritis.

To better understand the immunomodulatory effects of SerBut in CAIA, we analysed immune cell populations in the spleen and hock-draining LNs using flow cytometry. Our results showed that SerBut treatment increased Foxp3 expression in both CD4^+^ and CD8^+^ T cells in the spleen (Fig. 3h,i), indicating that SerBut induced expansion of systemic T_reg_ cells in the rheumatoid arthritis disease setting. In the hock-draining LNs (Fig. 3j), SerBut significantly reduced the proportion of T helper 17 (T_H_17) cells, as evidenced by the decreased percentage of RORγt^+^ CD4^+^ T cells. In addition, we observed a significant increase in interleukin (IL)-10-producing B cells within the hock-draining LNs (Fig. 3k), which play a vital role in maintaining immune homeostasis^42,46^. Finally, in the hock-draining LNs, SerBut treatment also mediated upregulation of Arg-1 expression in macrophages (Fig. 3l), resulting in an increased M2 (Arg-1^+^)/M1 (iNOS^+^) macrophage ratio (Fig. 3m). The shift towards a higher proportion of immunoregulatory M2-polarized macrophages suggests that SerBut promotes a more balanced immune response, which may be crucial for suppressing inflammation in the paws and reducing joint damage in the context of rheumatoid arthritis. Overall, these findings highlight the potential therapeutic utility of SerBut in treating rheumatoid arthritis by modulating the immune system at both the innate and adaptive compartments, mitigating disease symptoms.

**Fig. 4:**
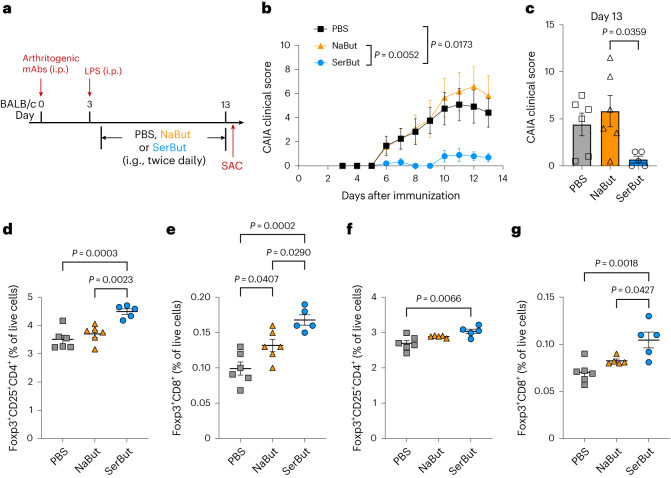
SerBut, but not NaBut, suppresses arthritis development. **a**, Experimental schema of the CAIA model. CAIA was induced by passive immunization with anti-collagen antibody cocktails on day 0, followed by i.p. injection of LPS on day 3. Starting on day 4, mice were orally gavaged with PBS (*n* = 6), NaBut (15 mg, molar equivalent to SerBut, *n* = 6) or SerBut (25 mg, *n* = 5) twice daily starting on day 4 until the end of the experiment. **b**, Arthritis scores in mice measured daily after immunization. **c**, Arthritis scores from PBS- or SerBut-treated mice on day 13. **d**,**e**, Percentage of Foxp3^+^CD25^+^ regulatory CD4^+^ T cells (**d**) or Foxp3^+^ regulatory CD8^+^ T cells (**e**) of live cells in the hock-draining LNs. **f**,**g**, Percentage of Foxp3^+^CD25^+^ regulatory CD4^+^ T cells (**f**) or Foxp3^+^ regulatory CD8^+^ T cells (**g**) of live cells in the spleen. The experiments comparing PBS and SerBut were repeated three times, and the results were consistent. NaBut was tested once as added in this experiment. Data represent mean ± s.e.m. Statistical analyses were performed using one-way ANOVA with Tukey’s post hoc test. *P* values less than 0.05 are shown. Source data

Next, we compared SerBut with free NaBut in CAIA and investigated whether initiating SerBut treatment post-disease induction (after the administration of the collagen antibody cocktail), with an increased dose frequency, remained efficacious in mitigating disease progression (Fig. 4a–c). This approach could be more clinically relevant for patients with rheumatoid arthritis who may have circulating autoantibodies for an extended period without showing symptoms^47^. We observed that NaBut did not demonstrate any therapeutic effect. Surprisingly, even during the shorter, 9-day treatment window, SerBut administration proved highly effective in preventing the onset of severe disease. Moreover, our data revealed that this SerBut treatment regimen also promoted the induction of T_reg_ cells in both the spleen and hock-draining lymph nodes (Fig. 4d–g). These results suggest that T_reg_ induction may play an essential role in the mechanism by which SerBut exerts its immunomodulatory effects in this model.

### SerBut suppresses EAE development via modulation of T cells and myeloid cells in the SC-dLNs

Multiple sclerosis is an autoimmune disease in which T cells are reactive to myelin autoantigens, resulting in a chronic demyelinating inflammation in the CNS. Evidence suggests a connection between gut microbiota and multiple sclerosis^26,27,48–52^, particularly dysbiosis in the gut microbiota of patients with multiple sclerosis, which includes a reduction of SCFA-producing bacteria^53^. Oral administration of SCFAs, such as butyrate, has been shown to alleviate the severity of EAE, a mouse model of multiple sclerosis, by reducing T_H_1 cells and increasing T_reg_ cells^28,54^. Butyrate has also been demonstrated to ameliorate demyelination and promote remyelination in a mouse model of cuprizone-induced demyelination^55^. Our biodistribution study revealed that SerBut significantly increased butyrate levels in the brain and spinal cord, in addition to the lymphatic tissues, suggesting its potential in treating multiple sclerosis.

**Fig. 5:**
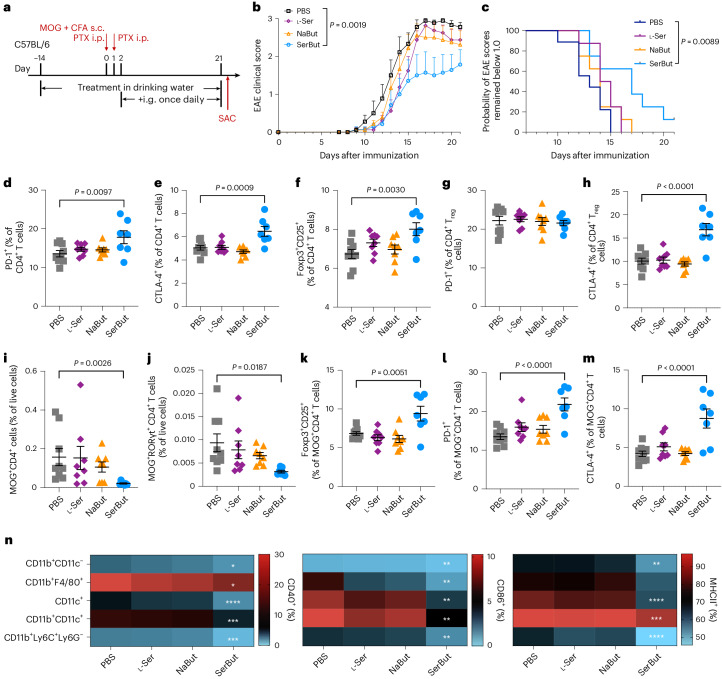
SerBut ameliorates EAE development more effectively than free butyrate or serine. **a**, Experimental schema. EAE was induced in C57BL/6 mice using MOG_35–__55_/CFA with pertussis toxin (140 ng). Mice were given drinking water containing 100 mM NaBut, l-Ser or SerBut from day −14 until the end of the study. On day 2 after EAE induction, PBS (*n* = 9), NaBut (15 mg, molar equivalent to SerBut, *n* = 8), l-Ser (12 mg, molar equivalent to SerBut, *n* = 8) or SerBut (24 mg, *n* = 7) was administered once daily. **b**, Disease progression as indicated by the clinical score. The areas under the curve were compared, and statistical analyses were performed using one-way ANOVA with Dunnett’s test. **c**, The probability of EAE clinical scores remaining below 1.0 for the four groups. Statistical analysis was performed using the log-rank (Mantel–Cox) test comparing every two groups. **d**–**m**, Phenotypes of CD4^+^ T cells from the SC-dLNs (that is, the iliac and cervical LNs), including the percentage of PD-1^+^ (**d**), CTLA-4^+^ (**e**) or Foxp3^+^CD25^+^ (**f**) of total CD4^+^ T cells; PD-1^+^ (**g**) or CTLA-4^+^ (**h**) of Foxp3^+^CD25^+^CD4^+^ T_reg_ cells; the percentage of MOG tetramer-positive CD4^+^ (**i**) or CD4^+^RORγt^+^ (**j**) T cells of total live cells; and Foxp3^+^CD25^+^ (**k**), PD-1^+^ (**l**) or CTLA-4^+^ (**m**) of MOG tetramer-positive CD4^+^ T cells. **n**, Heatmap of the percentage of co-stimulatory molecule (CD40^+^ or CD86^+^) or MHCII^+^ cells of myeloid cells in the SC-dLNs, indicated by the colour as shown in the corresponding scale bar. Data represent mean ± s.e.m. Experiments were repeated with similar, though not identical, dosing regimens, and the results were consistent. Statistical analyses were compared between PBS and each treatment group using one-way ANOVA with Dunnett’s test or Kruskal–Wallis test (if not normally distributed). In **b**–**m**, *P* values less than 0.05 are shown. In **n**, *P < 0.05, **P < 0.01, ***P < 0.001 and ****P < 0.0001. For raw figure and the *P* value for **n**, refer to Supplementary Fig. 10. Source data

We assessed the efficacy of SerBut in suppressing EAE development. To minimize physiological stress from daily oral gavage, mice were administered drinking water containing 100 mM SerBut for the 2 weeks preceding disease induction and were subsequently administered 24 mg SerBut via oral gavage once daily (Fig. 5a). The presence of NaBut or SerBut in the drinking water did not significantly impact the intake of water, as measured in healthy mice (Supplementary Fig. 7). SerBut treatment significantly reduced the EAE clinical score (Fig. 5b), indicating disease symptom alleviation. The treatment also delayed EAE onset, as indicated by the lower percentage of SerBut-treated mice with a disease score greater than 1 (Fig. 5c). Neither NaBut nor l-Ser at equimolar doses prevented disease development.

A thorough analysis of immune cell populations in the SC-dLNs showed that SerBut treatment significantly increased PD-1 and CTLA-4 expression on CD4^+^ T cells, expanded T_reg_ cells and upregulated CTLA-4 expression on CD4^+^ T_reg_ cells (Fig. 5d–h). Similarly, T_reg_ and PD-1 induction were also observed in CD8^+^ T cells (Supplementary Fig. 9). These results suggest that SerBut may help suppress excessive immune responses during EAE by modulating key immune checkpoint markers and T_reg_ cell populations. In addition, in the SC-dLNs, SerBut treatment reduced the number of myelin oligodendrocyte glycoprotein (MOG)-specific T cells and MOG^+^ RORγt^+^ CD4^+^ T cells (T_H_17 cells), antigen-specific pathogenic cells that contribute to EAE development (Fig. 5i,j). SerBut treatment also increased Foxp3, PD-1 and CTLA-4 expression on these MOG^+^ CD4^+^ T cells (Fig. 5k–m), potentially preventing these cells from promoting inflammation and tissue damage in EAE. Consistent with the disease readouts, neither NaBut nor l-Ser impacted these cellular immune responses.

In the innate immune compartment, SerBut treatment reduced the expression of the co-stimulatory markers, CD40 and CD86, as well as the antigen-presenting molecule MHC class II, on various myeloid cells including CD11b^+^CD11c^−^ cells, CD11b^+^F4/80^+^ macrophages, CD11c^+^ DCs, CD11b^+^CD11c^+^ DCs and CD11b^+^Ly6C^+^Ly6G^−^ myeloid-derived suppressor cells (MDSCs) (Fig. 5n and Supplementary Fig. 10). As these myeloid cells play a crucial role in initiating and propagating immune responses, the reduction of co-stimulatory molecules and MHC class II expression on these cells suggests that SerBut treatment may inhibit their activation and function. This could lead to a dampening of the inflammatory response and contribute to EAE suppression. MDSCs are reported to have immunosuppressive functions, such as inhibiting T cell proliferation and promoting T_reg_ expansion, in the context of EAE^56,57^. We observed not only a downregulation of activation markers on these cells but also a significant increase in the percentage of MDSCs in both SC-dLNs and mLNs (Supplementary Fig. 11). Overall, our results demonstrate that SerBut has a promising preventative effect on EAE by modulating the activity of various immune cell populations and reducing inflammatory responses. All these effects were observed exclusively with SerBut treatment and not with free l-Ser nor NaBut, suggesting the importance of serine conjugation in enhancing butyrate’s systemic and possibly CNS bioavailability.

### SerBut administered during the induction phase of EAE suppresses disease progression and inhibits immune responses in the spinal cord

We next sought to investigate whether administration of SerBut during the induction phase of EAE could also suppress disease progression and induce immunological changes in the spinal cord. In a preliminary, dose-optimization study, we compared SerBut administration via drinking water (150 mM) with oral gavage (48 mg per day). We observed that gavage was more effective in preventing the onset of EAE (Supplementary Fig. 13). We hypothesize that gavage treatment was more effective owing to its ability to ensure the complete delivery of the full dose, whereas the intake of SerBut-infused water may be more variable and subject to degradation in the stomach, reducing its bioavailability. This observation led us to exclusively use oral gavage for SerBut administration in subsequent EAE studies.

**Fig. 6:**
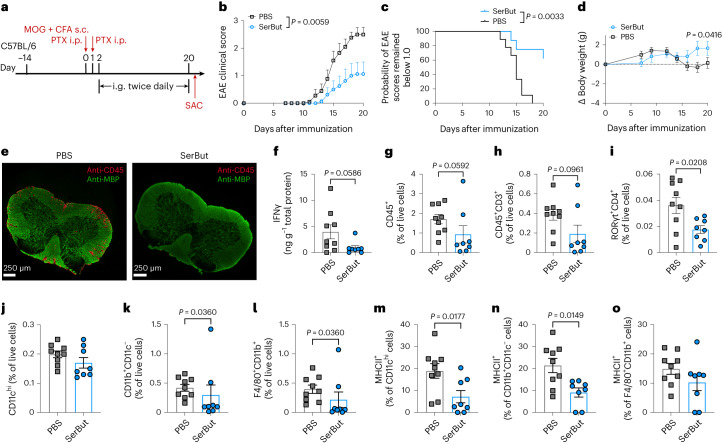
Twice-daily gavage of SerBut post-EAE induction suppressed disease progression. **a**, Experimental schema. EAE was induced in C57BL/6 mice using MOG_35–__55_/CFA with pertussis toxin (140 ng). On day 2 after EAE induction, PBS (*n* = 9) or SerBut (*n* = 8) at 24 mg per dose was administered twice daily by gavage. **b**, Disease progression as indicated by the clinical score. The areas under the curve were compared, and statistical analyses were performed using Student’s *t*-test. **c**, The probability of EAE clinical scores remaining below 1.0 in the two groups. Statistical analysis was performed using the log-rank (Mantel–Cox) test. **d**, Body weight changes. **e**, Representative immunofluorescence images of spinal cord sections from mice treated with PBS or SerBut. Red, anti-CD45 staining; green, anti-MBP staining. **f**, The concentration of IFNγ normalized by the total protein in the spinal cord homogenized supernatant. **g**–**l**, The percentage of CD45^+^ (**g**), CD45^+^CD3^+^ (**h**), RORγt^+^CD4^+^ (**i**), CD11c^hi^ (**j**), CD11b^+^CD11c^−^ (**k**) and F4/80^+^CD11b^+^ (**l**) of live cells in the spinal cord. **m**–**o**, The percentage of MHCII^+^ of CD11c^hi^ (**m**), CD11b^+^CD11c^−^ (**n**) or F4/80^+^CD11b^+^ (**o**) cells in the spinal cord. Data represent mean ± s.e.m. Experiments were repeated with similar, though not identical, dosing regimens, and the results were consistent. Statistical analyses were performed using Student’s *t*-test. Source data

In the following experiment, mice were administered with twice-daily gavage of SerBut or PBS after EAE induction (Fig. 6a). We observed that the SerBut-treated mice showed significantly lower EAE clinical scores compared with PBS-treated mice (Fig. 6b). Only two out of eight mice in the SerBut-treated group developed EAE with scores higher than one by the end of the study, and the rest remained healthy throughout the experiment (Fig. 6c). In addition, PBS-treated mice experienced a significant drop in body weight starting on day 14, whereas the SerBut-treated mice continued to grow over time (Fig. 6d).

When PBS-treated mice reached a plateau in disease score, we killed both PBS- and SerBut-treated mice and assessed the impact of treatment on the spinal cord. From immunofluorescence images with anti-CD45 and anti-myelin basic protein (MBP) staining, we observed substantial immune cell infiltration into the spinal cords of PBS-treated mice, but not in SerBut-treated mice (Fig. 6e and Supplementary Fig. 14). The two mice from the SerBut-treated group that did not respond to treatment also showed elevated immune cell infiltration into the spinal cord (Supplementary Fig. 14). In addition, we homogenized part of the spinal cord and measured IFNγ levels, noticing a decrease in IFNγ in the spinal cords of SerBut-treated mice (Fig. 6f).

We also quantified the effect of SerBut treatment on the immune cell compartment in the spinal cord using flow cytometry. We observed a decrease in immune cell populations across most immune cell types in the spinal cord, including CD45^+^ cells, CD3^+^ T cells and pathological T_H_17 cells (RORγt^+^ CD4^+^ T cells) (Fig. 6g–i), as well as macrophage-like CD11b^+^CD11c^−^ and CD11b^+^F4/80^+^ cells (Fig. 6j,k). However, no significant differences were detected in the frequencies of IL-17A^+^CD4^+^ and IFNγ^+^CD4^+^ cells between the PBS- and SerBut-treated groups. This observation is primarily attributed to the presence of non-responder mice in the treatment group and the low frequencies of these cell populations at the time of tissue collection (Supplementary Fig. 15). Given that IL-17A- and IFNγ-producing T cells are established benchmarks for T cell priming in EAE, future studies will refine the isolation and restimulation procedures to further analyse the impact of SerBut on these pathologically relevant T cell subsets^58,59^.

**Extended Data Fig. 1:**
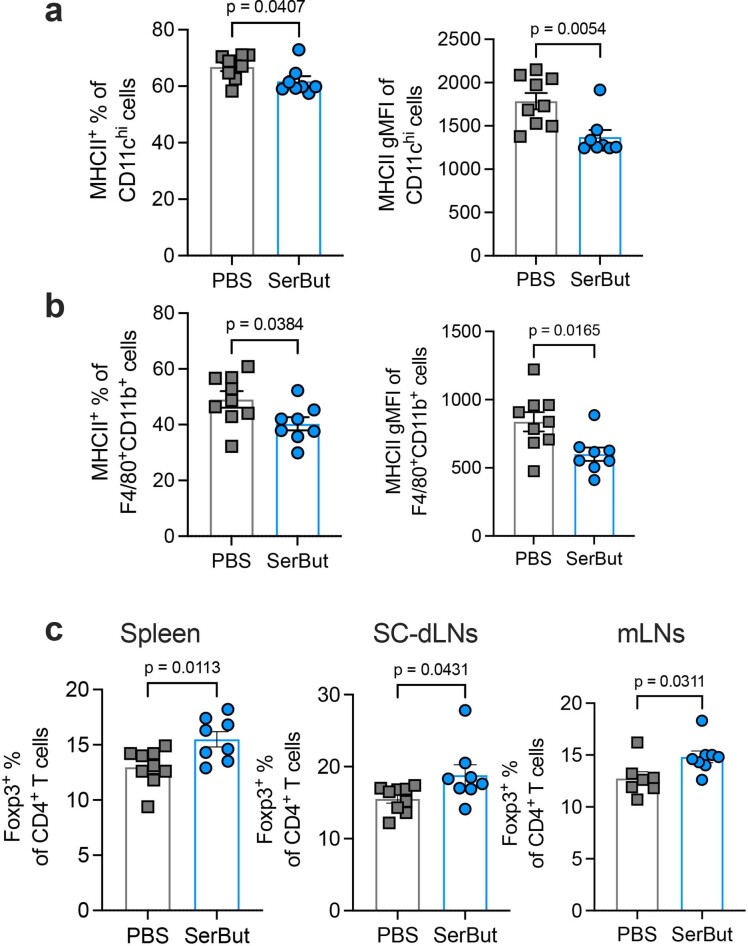
The immunological effects in peripheral tissues from SerBut treatment in the EAE model from the experiment in Fig. 6. **a,b**, The percentage of MHC class II^+^% and geometric mean fluorescent intensity (gMFI) of CD11chi dendritic cells (**a**) or F4/80^+^CD11b^+^ macrophages (**b**) isolated from mesenteric LN. **c**, The percentage of Foxp3^+^ of CD4^+^ T cells in the spleen, spinal cord-draining LNs (SC-dLNs), or mesenteric LNs (mLNs). Data represent mean ± s.e.m. Statistical analyses were performed using Student’s t-test. Source data

Although we did not observe a significant reduction in the CD11c^+^ DC population (Fig. 6j), we noted a decrease in MHC class II on their surface, as well as on CD11b^+^CD11c^−^ cells, indicating diminished activation of these cells in the spinal cords of SerBut-treated mice (Fig. 6m–o). The reduction of MHC class II on the myeloid cells was observed not only in the spinal cord but also in the mLNs (Extended Data Fig. 1a,b). In this experiment, we also observed that SerBut increased the frequency of Foxp3^+^ T_reg_ cells both systemically in the spleen and locally in the SC-dLNs and mLNs (Extended Data Fig. 1c), consistent with our findings in previous rheumatoid arthritis and EAE experiments. These results suggest that SerBut exerts its immunomodulatory effects both systemically and in disease-related tissues.

**Extended Data Fig. 2:**
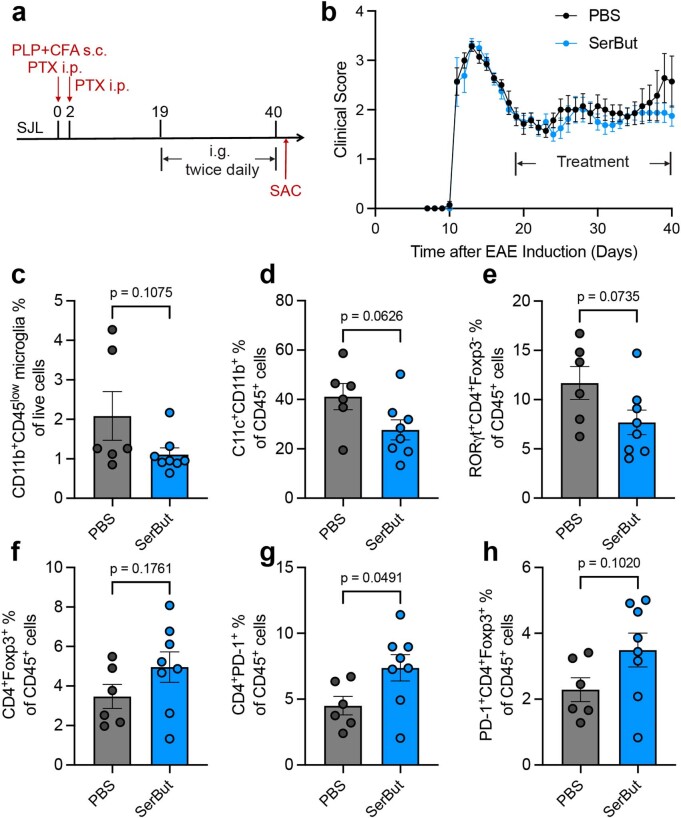
SerBut did not exhibit significant therapeutic efficacy in relapsing-remitting EAE. **a**. Experimental schema. EAE was induced in SJL/J mice using PLP_139-151_/CFA with 100 ng pertussis toxin. Starting on day 19, mice were regrouped into two treatment groups with equivalent average clinical score, and received twice daily oral gavage of either PBS (*n* = 6) or SerBut (*n* = 8) at 24 mg/dose. **b**. Disease progression as indicated by the clinical score. **c**. The percentage of CD11b^+^CD45^low^ microglia cells of live cells in the spinal cord. **d-h**. The percentage of CD11b^+^CD11c^+^(d), RORγ^+^CD4^+^Foxp3^−^(e), CD4^+^Foxp3^+^(f), CD4^+^PD-1^+^(g), PD-1^+^CD4^+^Foxp3^+^(h) of CD45^+^ cells in the spinal cord. Data represent mean ± s.e.m. Statistical analyses were performed using Student’s t-test. Source data

We next used the proteolipid protein (PLP)/Complete Freund’s Adjuvant (CFA)-induced EAE relapsing–remitting model to investigate the therapeutic potential of SerBut administration in EAE (Extended Data Fig. 2a). For this purpose, we began treating the mice with PBS or SerBut on day 19 after they had recovered from the first wave of disease. SerBut treatment did not significantly impact initial relapse onset compared with PBS-treated mice (Extended Data Fig. 2b), potentially owing to the relatively short duration of treatment. Despite this, with continued SerBut administration, the clinical scores in SerBut-treated mice remained stable, whereas some PBS-treated mice entered a second relapse phase, albeit with considerable variation. No significant differences were observed in clinical scores between the two groups, which might be attributed to the advanced stage of disease and limited duration of treatment. Intriguingly, flow cytometric analysis of the spinal cord showed a significant upregulation of PD-1 expression on both CD4^+^ and CD8^+^ T cells (Extended Data Fig. 2g and Supplementary Fig. 16). While these findings are preliminary, they suggest the potential of SerBut in modulating severe neuroinflammation and merit further model modification and mechanistic studies.

### SerBut does not impact global immune responses to vaccination or alter blood chemistry toxicological markers

Several immune modulators used in autoimmunity blunt systemic immune responses to vaccination or infection. As a benchmark, we used fingolimod (FTY720), a Food and Drug Administration-approved oral compound for treating multiple sclerosis^60^. FTY720 targets the sphingosine-1-phosphate receptor and modulates the immune system by sequestering lymphocytes in lymph nodes, reducing their migration to the CNS and ultimately lowering inflammation^61^. Studies have shown that prophylactic oral administration of fingolimod completely prevents the development of EAE in mice, while therapeutic administration reduces the severity of EAE^61^. However, FTY720 treatment can inhibit global immune responses, as demonstrated by our earlier work, where FTY720 administrated to mice vaccinated with ovalbumin (OVA) and adjuvant reduced the generation of OVA-specific immunoglobulin G (IgG)^62^.

**Fig. 7:**
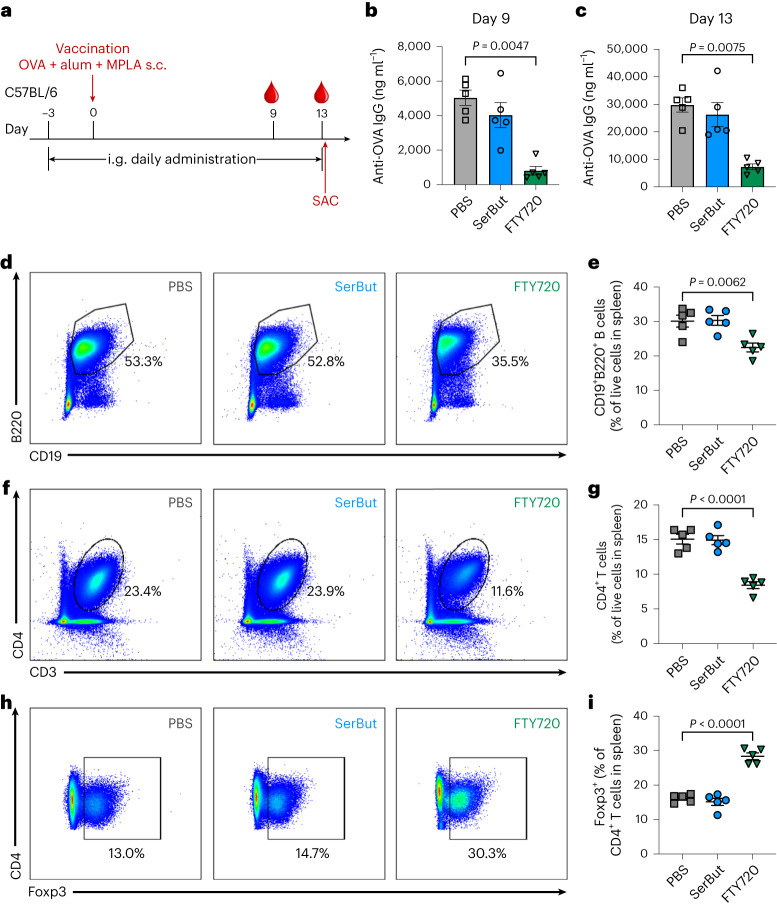
SerBut does not impact immune responses to vaccination compared with FTY720. **a**, Experimental schema. Mice were orally gavaged with PBS, SerBut (twice daily, 25 mg per dose) or FTY720 (once daily, 0.02 mg per dose) starting on day −3 until the end of the experiment. On day 0, mice were immunized subcutaneously in the front hocks with 10 μg endotoxin-free OVA, 50 μg alum and 5 μg MPLA. **b**,**c**, Mice were bled on day 9 (**b**) and day 13 (**c**), and plasma was analysed for anti-OVA IgG antibodies. **d**–**i**, Representative flow cytometry dot plots of CD19^+^B220^+^ (**d**,**e**), CD4^+^ (**f**,**g**), and Foxp3^+^CD4^+^ T cells (**h**,**i**) in the spleen, along with their respective percentages of the parental cell population (**d**,**f**,**h**), or of the total live cells (**e**,**g**,**i**). *n* = 5 mice per group. Data represent mean ± s.e.m. Statistical analyses were performed using one-way ANOVA with Dunnett’s post hoc test. *P* values less than 0.05 are shown. Source data

**Extended Data Fig. 3:**
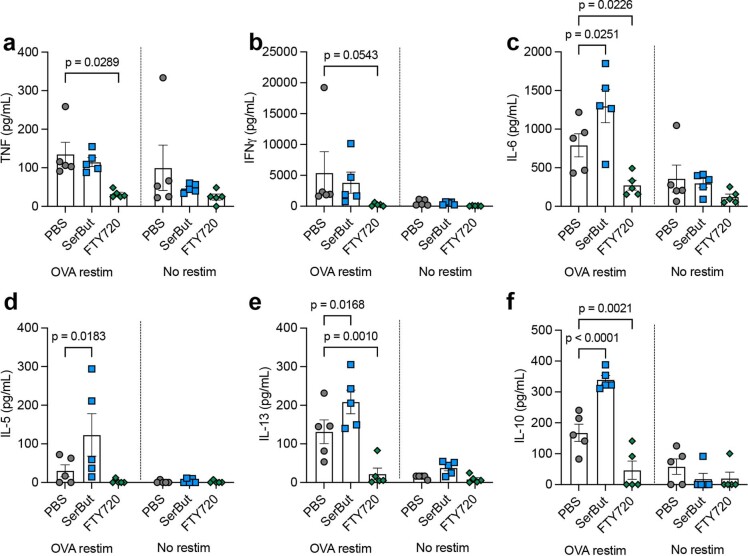
SerBut’s impact on immune responses to vaccination in comparison to FTY720. Production of cytokines TNFa (**a**), IFNγ (**b**), IL-6 (**c**), IL-5 (**d**), IL-13 (**e**), and IL-10 (**f**), measured in the supernatant of isolated splenocytes upon restimulation with OVA protein for 3 days. *n* = 5 mice per group. Data represent mean ± s.e.m. Statistical analyses were performed using two-way ANOVA with Dunnett’s post hoc test. P values less than 0.05 were shown. Source data

To gain insight into whether SerBut impacts immune responses to vaccination, we subcutaneously vaccinated naive mice in the hocks with OVA, in combination with the adjuvants alum and monophosphoryl-lipid A (MPLA), which mimics the clinical vaccine adjuvant AS-04. Mice were treated with either PBS, SerBut or FTY720 by oral gavage (Fig. 7a). Blood collected on days 9 and 13 revealed OVA-specific IgG antibody generation in both PBS- and SerBut-treated mice but much less so in FTY720-treated mice, indicating that SerBut does not influence IgG antibody generation, unlike FTY720, in response to OVA vaccination (Fig. 7b,c). To evaluate cellular responses, we killed the mice and isolated immune cells from hock-draining LNs. FTY720 reduced B cell (CD19^+^B220^+^), T cell (CD3^+^) and CD4^+^ T cell populations in both hock-draining LNs and the spleen (Fig. 7d–g and Supplementary Fig. 20a–c). Interestingly, FTY720 substantially increased Foxp3 T_reg_ cells in both LNs and the spleen (Fig. 7h,i and Supplementary Fig. 20d). By contrast, SerBut showed no impact on any of these cell populations in OVA-vaccinated naive mice. When we restimulated splenocytes isolated from mice with OVA in vitro, we observed a significant reduction in cytokine production, including TNF, IFNγ, IL-6, IL-5, IL-13 and IL-10 (Extended Data Fig. 3), indicating that FTY720 suppressed antigen-specific T cell responses to OVA. By contrast, SerBut treatment did not suppress these cellular responses, although, interestingly, we did observe some significant increases in IL-6, IL-5, IL-13 and IL-10 production with SerBut treatment.

To determine the impact of SerBut administration on organ function, we also conducted biochemistry analysis on serum from SerBut-treated mice (Supplementary Fig. 21). Overall, we did not observe significant changes in markers of liver, pancreas and kidney toxicity following SerBut treatment, while FTY720 induced a modest reduction in blood urea nitrogen levels. Taken together, these findings suggest that SerBut does not adversely impact global immune responses to vaccination and is safe for mice when administered via twice-daily oral gavage.

### SerBut induces less pronounced immunological effects on healthy mice than mice with an inflammatory insult

**Extended Data Fig. 4:**
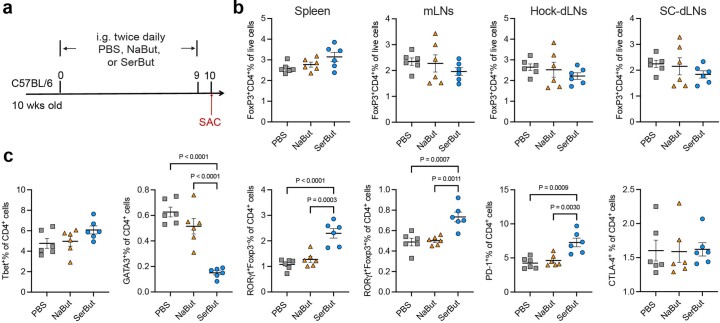
Immunological effects of SerBut treatment in healthy C57BL/6 mice on the T cells in the spleen, LNs, or lamina propria. **a**. Experimental schema. C57BL/6 mice at 10 wks old were oral administered with PBS (*n* = 5), NaBut (15 mg, molar equivalent to SerBut, *n* = 6) or SerBut (25 mg, *n* = 6), twice daily for 10 days, and sacrificed for cellular analysis. **b**. CD4^+^Foxp3^+^ Tregs percentage of live cells in spleen, mesenteric LNs (mLNs), hock-draining LNs (hock-dLNs), and spinal cord-draining LNs (SC-dLNs). **c**. Tbet^+^, GATA3^+^, RORγ^+^ Foxp3^−^, RORγ^+^ Foxp3^+^, PD-1^+^, CTLA-4^+^ of CD4^+^ T cells in the spleen. Data represent mean ± s.e.m. Statistical analyses were performed using one-way ANOVA with Tukey’s post hoc test. P values less than 0.05 were shown. Source data

To further investigate the potential impact of SerBut on non-disease-bearing mice, we administered PBS, NaBut or SerBut to healthy C57BL/6 mice via twice-daily oral gavage and measured the immunological impact on various tissues by flow cytometry. Notably, unlike in autoimmune disease models (CAIA and EAE), SerBut treatment did not elicit CD4^+^ T_reg_ induction in the spleen or lymph nodes of healthy mice (Extended Data Fig. 4b). These results suggest that SerBut may selectively induce T_reg_ cells in inflammatory contexts rather than in healthy physiology.

Interestingly, in the spleen, SerBut treatment induced a marked decrease in T_H_2 cells and an increase in T_H_17 cells (Extended Data Fig. 4c). The augmentation of T_H_17 cells in healthy mice following SerBut administration is particularly intriguing, as it contrasts the reduction of T_H_17 cells induced by SerBut in the CAIA and EAE models, which are characterized by overabundant, T_H_17-biased immune responses. This unexpected expansion in T_H_17 cells warrants further exploration; however, it is worth noting that SerBut administration has also mediated the expansion of RORγt^+^ T_reg_ cells, a distinct population of T_reg_ cells known for their enhanced suppressive capacity compared with Foxp3^+^RORγt^−^ T_reg_ cells^63^. Moreover, we observed an upregulation of PD-1 expression on CD4^+^ T cells, whereas CTLA-4 expression remained unchanged (Extended Data Fig. 4c). These findings in healthy mice suggest that differential regulatory pathways may be involved in the modulation of T cell exhaustion markers by SerBut.

In the lamina propria of healthy mice, we also did not observe T_reg_ induction following SerBut treatment (Supplementary Fig. 22). However, SerBut administration reduced the frequency of RORγt^+^ Foxp3^−^ T_H_17 cells in the lamina propria, presenting an intriguing contrast to its impact on the splenic immune response. The disparate effects of SerBut on T_H_17 cell populations in the spleen versus the lamina propria could be influenced by several factors. The spleen, as a system-wide secondary lymphoid organ, is broadly pivotal in immune cell activation and proliferation. In this context, SerBut, which was distributed systemically and at a high concentration in the spleen, may modulate immune responses, potentially by influencing antigen-presenting cells (APCs) or altering the T cell responses—suppressing T_H_2 responses while favouring T_H_17 differentiation, as evidenced by the decreased GATA3^+^ T cells and increased RORγt^+^ T cells (Extended Data Fig. 4c). By contrast, the lamina propria, primarily isolated from the lower gastrointestinal tract in our study, is a site where immune responses are stringently regulated to prevent unnecessary inflammation against commensal bacteria, particularly in healthy states^64^. The observed reduction of T_H_17 cell numbers there could be indicative of SerBut’s potential role in promoting a more tolerogenic environment, particularly in the presence of stimuli from the microbiome^65,66^. This could involve the inhibition of local inflammatory signals that could differentially affect T_H_17 responses when compared with the spleen. In addition, the observed tissue-specific difference and contextual variations between healthy and disease states may be indicative of unique T cell trafficking patterns. Under normal conditions, T_H_17 cells are enriched within mucosal tissues such as lamina propria and can recirculate to the spleen. However, in the context of severe tissue inflammation, these T_H_17 cells may contribute to tissue and organ damage^67^. In such scenarios, SerBut could potentially inhibit these pathways.

Furthermore, our findings indicate that SerBut’s impact may be more systemic than that of NaBut, which showed no notable changes upon administration in healthy mice. In the mLNs, SerBut did not alter myeloid cell activation markers, except for a decrease in MHC class II expression on macrophages (Supplementary Fig. 23). This lack of impact on myeloid cells contrasts with observations in the CAIA and EAE models and may be attributed to the fact that cells are less activated during normal physiological conditions. Overall, the impact of SerBut on immune cell populations in healthy mice was minimal and tissue dependent, particularly regarding the T_reg_ and myeloid compartments, where the significant effects seen in the CAIA and EAE models were absent. The lack of immunomodulation in healthy mice suggests a potentially favourable profile for SerBut, as it may reduce the risk of unintended immunosuppression in non-diseased states.

## Discussion

In this study, we developed a prodrug, SerBut, by conjugating butyrate with l-Ser and investigated its potential in preventing and treating rheumatoid arthritis and multiple sclerosis using CAIA and EAE as mouse models. Orally administered free butyrate can be absorbed and quickly metabolized by host tissues and cells via butyryl-CoA/acetate CoA transferase and phosphotransbutyrylase–butyrate kinase pathways^68^. In the colon, butyrate is primarily consumed by the colonocytes as an energy source^29^. By using a simple chemical strategy to conjugate serine to butyrate, we improved the oral bioavailability of butyrate including into the CNS, engineering a prodrug candidate that offers several advantages, including a lack of unpleasant odour and taste and higher efficacy compared with free butyrate. Our results demonstrated that SerBut substantially ameliorated the severity of both diseases, modulated key immune cell populations and reduced inflammatory responses, all without compromising global immune responses to vaccination.

By conjugating with l-Ser, an essential amino acid that is readily absorbed through transporters in the gastrointestinal tract, SerBut significantly enhanced plasma butyrate concentrations compared with NaBut within 30 min post-administration (Supplementary Fig. 4). Upon entering the bloodstream, SerBut is likely to encounter hydrolytic enzymes capable of cleaving the ester bond to release butyrate. These enzymes are present in various tissues^69,70^, with particularly high concentrations in the (1) liver, owing to its abundance of esterases and proteases, which relate to its pivotal role in systemic drug metabolism, (2) plasma, where circulating esterases can act on a wide variety of substrates, and (3) organs such as the kidneys, which have high blood flow and substantial enzymatic activity. Furthermore, the serine moiety could be recognized by amino acid transporters on cell membranes, enhancing cellular uptake before intracellular enzymes catalyse the release of butyrate. Consequently, we observed enhanced butyrate levels across multiple major organs, including the spinal cord and brain, in SerBut-treated mice compared with those treated with NaBut. It is important to clarify that the dose used in our biodistribution study was acutely administered at approximately 15 times higher than the single dose used in the subsequent disease model efficacy studies. This was to ensure the detectable accumulation of butyrate in various tissues and organs, including the CNS, using liquid chromatography with tandem mass spectrometry (LC-MS/MS) methods. The therapeutic dose used later was markedly lower and within a range that did not yield detectable butyrate levels in our LC-MS/MS analysis, nor did it elicit any observable side effects or behavioural changes in the mice.

Butyrate, a key metabolite from commensal bacteria, has multiple modulating effects across different types of immune cells. It has been shown to facilitate peripheral generation of T_reg_ cells through both direct upregulation of Foxp3 by HDAC inhibition^16,17^ and indirect effects from the induction of tolerogenic DCs^19,71^. In our study, we demonstrated that SerBut treatment increased peripheral T_reg_ cells in both CAIA and EAE settings, as well as in both local draining LNs and spleen. Notably, we observed enhanced biodistribution of SerBut in these tissues after oral administration, which is not limited to gut tissues as previously reported^72,73^. Butyrate has also been shown to modulate the differentiation of macrophages and DCs. Through HDAC inhibition, butyrate downregulates LPS-induced pro-inflammatory cytokines produced by macrophages^34^ and activates macrophage metabolism towards oxidative phosphorylation through upregulation of Arg-1, promoting an anti-inflammatory M2 phenotype^74^. In our CAIA model, enhancing the systemic bioavailability of butyrate through SerBut significantly increased the M2/M1 macrophage ratio in hock-draining LNs, which could exert direct anti-inflammatory effects on paw and joint inflammation. Moreover, butyrate has been shown to suppress DC activation and induce tolerogenic DCs through a combination of signalling via GPR109A and HDAC inhibition^19^. In our study, we observed that SerBut suppressed LPS-induced activation, including the co-stimulatory markers CD80 and CD86, and MHC class II expression of BMDCs isolated from mice compared with NaBut. Consistently, in the prophylactic EAE model, we observed downregulation of these co-stimulatory markers and MHC class II on various myeloid cells across different tissues, suggesting the important roles that butyrate can play in suppressing disease progression through this pathway while delivered systemically.

Myeloid cells, such as DCs and macrophages, play a crucial role in antigen presentation and T cell activation. For instance, CD86 interacts with CD28 on T cells, promoting their activation and proliferation^75,76^. CTLA-4 is a surface receptor predominantly expressed on T cells, particularly on T_reg_ cells, and competes with CD28 for binding to CD86 on myeloid cells, delivering an inhibitory signal to T cells^77^. Intriguingly, in our EAE experiment, we observed that SerBut treatment led to a significant increase in CTLA-4 expression on both total CD4^+^ T cells and T_reg_ cells in the SC-dLNs. This concurrent upregulation of CTLA-4 on T cells and downregulation of CD86 on myeloid cells can synergistically contribute to a more profound suppression of immune activation, ultimately dampening autoimmune responses.

In the prophylactic EAE experiment, we demonstrated that twice-daily administration of SerBut significantly reduced immune cell infiltration into the spinal cord. Future studies will explore whether these effects on the CNS effect are modulated directly by butyrate that crosses the BBB or indirectly owing to butyrate’s effects on peripheral immune cells that subsequently induce immunomodulation in the CNS. In addition, investigating the impact of butyrate on the BBB (or the blood–spinal cord barrier) endothelial cell functions and barrier integrity is of further interest. Distinctive effects of SerBut were more pronounced when administered to C57BL/6 mice in the MOG/CFA model, either starting before or during the EAE induction phase. By contrast, in the PLP-EAE relapsing–remitting model on SJL/J mice, when treatment was initiated after peak disease severity, SerBut did not yield significant therapeutic benefits. It is important to note that the SJL/J and C57BL/6 EAE models show differences in the distribution of inflammatory and demyelinating lesions: SJL mice generally show more extensive brain inflammation, while C57BL/6 mice develop more severe spinal cord damage than brain damage^78–80^. We have previously observed higher butyrate levels in the spinal cord compared with the brain following oral SerBut administration, prompting the hypothesis and need for further studies on butyrate’s direct effects at sites of inflammation.

Interestingly, we noticed that SerBut’s immunomodulatory effects are context dependent, promoting immune homeostasis in inflammatory settings. This is evidenced by our findings that SerBut induces T_reg_ induction in the autoimmune disease models such as CAIA and EAE (Figs. 3h,i, 4d–g and 5f,k, Extended Data Fig. 1c and Supplementary Fig. 9), but not following administration in healthy mice or in OVA-vaccinated mice (Fig. 7h, Extended Data Fig. 4b, and Supplementary Figs. 20d and 22a,b). These differences might be attributed to the distinct immunological environments present under these conditions. In autoimmune settings, the inflammatory milieu, characterized by antigenic or antibody stimuli and cytokine production, may be more conducive to the conversion of naive T cells into T_reg_ cells in response to SerBut treatment. We observed similar context-dependent differences in T_H_17 responses: SerBut treatment in disease models with T_H_17-biased autoimmunity induced a reduction in T_H_17 cells following treatment (Figs. 3j, 5j and 6i, and Extended Data Fig. 2e), while in healthy mice, the impact of SerBut administration was more nuanced and tissue-specific (Extended Data Fig. 4c and Supplementary Fig. 22c,d). Moreover, the impact of SerBut on myeloid cells was also more pronounced in the presence of inflammation (Figs. 3l,m, 5n and 6m–o, Extended Data Fig. 1a,b, and Supplementary Figs. 10 and 11) than in healthy mice, where myeloid cells were not pre-activated (Supplementary Fig. 23). The differential, disease-dependent immunological effects of SCFAs are corroborated by other literature across various fields, including cancer immunotherapy and vaccine responses^81,82^. In the case of protein vaccination with the adjuvant MPLA mixed with alum (analogous to AS04, used in licensed vaccines), immune responses were not suppressed.

We have shown that SerBut has several potential benefits over free NaBut for clinical translation: serine conjugation to butyrate masks its odour and taste, yields higher bioavailability and shows greater efficacy in suppressing autoimmune disease models compared with currently available NaBut. Although various studies have demonstrated the efficacy of NaBut, for example, in suppressing arthritis in mouse models, it is typically administered in food or in drinking water at high concentrations over a 5–8-week period^83–86^. In our experiment comparing NaBut and SerBut’s efficacies in an arthritis model (Fig. 4), we used a lower dose of NaBut (15 mg, twice-daily oral gavage) over a shorter period (10 days), resulting in no observed therapeutic effects. By contrast, SerBut, administered at an equivalent butyrate dose, showed superior efficacy in suppressing arthritis. The dosage used in our studies can be converted to 6 g of SerBut per human dose, which could be conveniently formulated as a soluble powder for daily water consumption. High dosages or extended dosing regimens, such as those used with NaBut in the literature, may pose considerable challenges for clinical application owing to factors such as the foul odour and taste when consumed as drinking water, as well as the impracticality of encapsulating large quantities. Therefore, SerBut offers a more viable solution by addressing these challenges associated with NaBut while also demonstrating enhanced therapeutic potential.

Importantly, SerBut did not adversely impact global immune responses to vaccination, as demonstrated by the generation of equivalent OVA-specific IgG humoral and cellular immune responses in both PBS- and SerBut-treated mice. Although we observed effective immune modulation in the context of autoimmune arthritis and EAE, the immune response elicited by a strong, T_H_1-biasing adjuvant (the TLR4 agonist MPLA in the alum depot) during vaccination was sufficient to overcome the effects of SerBut^87^. This distinction offers a compelling insight into the potential of SerBut as a more targeted therapeutic strategy for chronic autoimmune diseases, with a possibly lower risk of the adverse effects associated with broad immunosuppression.

Our study provides evidence that SerBut has potential as a next-generation therapeutic agent for rheumatoid arthritis and multiple sclerosis. Further studies, including preclinical and clinical studies, are needed to better understand the long-term safety and efficacy of SerBut in the context of rheumatoid arthritis, multiple sclerosis, and other autoimmune and inflammatory diseases. Given its broad immunomodulatory effects shown in our study, it would be valuable to explore the potential of SerBut in treating a broader range of immune-related conditions.

## Methods

### Study design

The objective of this study was to chemically conjugate l-Ser to butyrate to improve butyrate’s oral bioavailability and investigate the therapeutic potential of the conjugate, SerBut, in the context of rheumatoid arthritis and multiple sclerosis using CAIA and EAE mouse models.

In the biodistribution study, we quantified the butyrate content in various major organs following oral gavage of SerBut. In the rheumatoid arthritis model, mice were treated daily with either PBS, NaBut or SerBut via oral gavage. Paw inflammation was assessed over time, and the pathology of inflamed paws and joints was evaluated using histology. Immune cell phenotypes were analysed by flow cytometry at the end of the experiment when PBS-treated mice reached a plateau in disease scores. In the EAE model, we compared the efficacy of SerBut, PBS, free butyrate and free l-Ser in preventing disease progression. Immune responses were evaluated at the end of the experiment when PBS-treated mice reached a plateau in disease scores. In the vaccination study, the global immunosuppressive effects of SerBut were compared with those of FTY720. The study endpoint was determined based on previous reports that day 13 after OVA immunization was sufficient to induce anti-OVA IgG antibodies^62^.

Sample size was determined using results obtained from previous and preliminary studies. At least five, and in most cases seven to nine, independent biological replicates were examined for each group analysed. See figure legends for details on *n* for each figure. All experiments were replicated at least twice. Mice were randomly assigned to treatment groups, except in the PLP-EAE study where mice with already-established disease were assigned to treatment groups based on average clinical score. The person assessing clinical scores for rheumatoid arthritis and EAE experiments was different from the person administering treatment and was blinded to the treatment group. The person performing fluorescence imaging and histology analysis was also blinded to treatment groups. Statistical methods are described in the ‘Statistical analysis’ section.

### Synthesis of SerBut

l-Ser (20 g, 0.19 mol) was added to trifluoroacetic acid (200 ml), and the suspension was stirred for 30 min until everything dissolved. Butyryl chloride (25.7 ml, 0.23 mol) was then added to the solution and the mixture was stirred for 2 h at room temperature. The reaction was then transferred to an ice bath and diethyl ether (500 ml) was added, which resulted in a precipitation of a white solid. The resultant fine white precipitate was collected by filtration, washed with cold diethyl ether and dried under vacuum to afford 26.3 g SerBut (0.15 mol, 79%). The final product was confirmed by ^1^H NMR (500 MHz, DMSO-*d*_6_) (ppm): 0.88 (3H, t), 1.55 (2H, m), 2.32 (2H, t), 4.30 (1H, t), 4.43 (2H, d), 8.66 (2H, s), 14.06 (1H, s).

### Mice

C57BL/6 mice, aged 8–12 weeks, were purchased from Charles River (strain code 027). BALB/c mice, aged 6–10 weeks, were purchased from the Jackson Laboratory (strain code 000651). SJL/JCrHsd mice, aged 6 weeks, were purchased from Envigo (strain code 052). C57BL/6, BALB/c and SJL/JCrHsd mice were maintained in a specific pathogen-free facility at the University of Chicago. Mice were maintained on a 12 h light/dark cycle at a room temperature of 20–24 °C. All protocols used in this study were approved by the Institutional Animal Care and Use Committee of the University of Chicago.

### Flow cytometry and antibodies

Flow cytometry was performed using BD LSRFortessa, and data were analysed using FlowJo version 10.8.0. Antibodies against the following markers were used in the rheumatoid arthritis and EAE mouse models: iNOS (BUV737, catalogue number 367-5920-82, Invitrogen), CD11c (BV421, catalogue number 562782, BD Biosciences), CD11c (BV785, catalogue number 563735, BioLegend), Ly6C (BV605, catalogue number 128036, BioLegend), CD11b (BV650, catalogue number 563402, BD Biosciences), CD11b (BV711, catalogue number 101242, BioLegend), Ly6G (AF488, catalogue number 127626, BioLegend), CD40 (PerCP/Cy5.5, catalogue number 124624, BioLegend), CD40 (BUV615, catalogue number 751646, BD Biosciences), CD206 (PE, catalogue number 141706, BioLegend), CD206 (AF700, catalogue number 141734, BioLegend), Arginase 1 (PE-Cy7, catalogue number 25-3697-82, Invitrogen), F4/80 (APC, catalogue number 123116, BioLegend), F4/80 (PE, catalogue number 565410, BD Biosciences), CD86 (AF700, catalogue number 105024, BioLegend), CD86 (BUV395, catalogue number 564199, BD Biosciences), I-A/I-E (APC/Cy7, catalogue number 107628, BioLegend), I-A/I-E (BV421, catalogue number 107632, BioLegend), CD19 (BUV396, catalogue number 563557, BD Biosciences), CD3 (BUV737, catalogue number 741788, BD Biosciences), CD3 (BV605, catalogue number 100351, BioLegend), CD3 (APC-Fire750, catalogue number 100362, BioLegend), CD4 (BV605, catalogue number 100548, BioLegend), CD4 (BV711, catalogue number 100550, BioLegend), CD4 (BUV496, catalogue number 612952, BD Biosciences), CD4 (AF647, catalogue number 553051, BD Biosciences), PD-1 (BV711, catalogue number 135231, BioLegend), PD-1 (APC-Cy7, catalogue number 135223, BioLegend), Foxp3 (AF488, catalogue number 53-5773-82, Invitrogen), PD-L1 (BV711, catalogue number 563369, BD Biosciences), RORγt (PerCP/Cy5.5, catalogue number 562683, BD Biosciences), RORγt (APC, catalogue number 562682, BD Biosciences), RORγt (BV421, catalogue number 562894, BD Biosciences), CD5 (PE, catalogue number 100607, BioLegend), CTLA-4 (PE-Cy7, catalogue number 25-1522-80, Invitrogen), CTLA-4 (PE-Cy7, catalogue number 106314, BioLegend), CD25 (APC, catalogue number 162105, BioLegend), CD25 (PerCP/Cy5.5, catalogue number 561112, BD Biosciences), CD25 (BV650, catalogue number 10238, BioLegend), CD8 (AF700, catalogue number 100730, BioLegend), CD8 (BUV737, catalogue number 612759, BD Biosciences), IL-10 (APC/Cy7, catalogue number 505036, BioLegend), CD45 (BUV395, catalogue number 564279, BD Biosciences), CD45 (V450, catalogue number 560501, BD Biosciences) and CD45 (BUV805, catalogue number 748370, BD Biosciences). The I-A(b) mouse MOG 38-49 GWYRSPFSRVVH (MOG tetramer, PE) was obtained from NIH Tetramer Core Facility.

### In vitro histone acetylation assay

Raw 264.7 macrophages were cultured to 50% confluency before stimulation with the indicated concentration of inhibitor with 100 ng ml^−1^ LPS (Invivogen tlrl-smlps) and cultured for 18 h. After stimulation, cells were collected via scraping before histone isolation according to the manufacturer’s protocol (Abcam 113476). Protein gels were then loaded with 8 μg of total protein isolate as quantified via bicinchoninic acid assay (Pierce 23225) before semi-dry transfer. The membrane was blocked with 5% BSA in PBS-T before probing with anti-total H3 (CST 4499) and anti-acetylated H3K9 (CST 9649) at 1:1,000 in 2% BSA in phosphate-buffered saline with Tween 20, followed by the HRP-conjugated secondary antibody (Southern Biotech 4050-05) at 1:10,000 in 2% BSA in PBS-T and detection.

### Mouse BMDC isolation and activation study

Mouse BMDCs were collected from 6-week-old female C57BL/6 mice, as described by ref. ^88^. BMDCs were seeded at 3 × 10^6^ total cells per plate in petri dishes. Cells were cultured at 37 °C and 5% CO_2_ in the media: RPMI 1640 (Life Technologies), 10% HIFBS (Gibco), GM-CSF (20 ng ml^−1^; recombinant mouse GM-CSF (carrier-free) from BioLegend), 2 mM l-glutamine (Life Technologies) and 1% antibiotic–antimycotic (Life Technologies). Media were replenished on days 3 and 6. Cells were used on day 9. Isolated BMDCs were plated in round-bottom 96-well plates at 100,000 cells per well in RPMI media and co-cultured with different concentrations of either NaBut or SerBut (from 0.02 mM to 1.8 mM) for 24 h. Subsequent to addition of butyrate compounds, cells were stimulated with LPS (1 μg ml^−1^) for another 18 h. The supernatant of cell culture was collected and analysed by LEGENDplex to analyse the concentrations of cytokines (BioLegend). BMDCs were collected and stained with Live/Dead dye (catalogue number L34957, Invitrogen) and fluorescent antibodies against CD11c (PE-Cy7, catalogue number 558079, BD Biosciences), MHC class II (APC-Cy7, catalogue number 107628, BioLegend), CD80 (PE, catalogue number 104708, BioLegend) and CD86 (FITC, catalogue number MA1-10300, Invitrogen). Cell phenotype was analysed using flow cytometry (BD LSRFortessa).

### Biodistribution of SerBut

C57BL/6 mice were orally administered with 50.4 mg NaBut or 80 mg SerBut (both containing equivalent 40 mg butyrate). At 3 h post-administration, mice were anaesthetized under isoflurane and blood was collected via cheek bleeding, and mice were then transcardially perfused with a minimum of 30 ml PBS containing 1 mM EDTA. Organs, including the liver, mLNs, spleen, lung, spinal cord and brain, were collected, immediately frozen in dry ice and then transferred to −80 °C until further processing.

To extract butyrate from plasma or organs, a 1:1 v/v acetonitrile (ACN) to water solution was used. Plasma was mixed 1:1 with the ACN/water solution and centrifuged to remove denatured proteins. Organs were weighed, transferred to Lysing Matrix D tubes and combined with the 1:1 v/v ACN/water solution. Samples were then lysed using a FastPrep-24 5 G homogenizer (MP Biomedicals) and centrifuged. The supernatants were collected for butyrate measurement.

Samples were prepared and derivatized, as described previously^7,89^. A 3-nitrophenylhydrazine (NPH) stock solution was prepared at 0.02 M in water:ACN 1:1 v/v. A 1-ethyl-3-(3-dimethylaminopropyl)carbodiimide (EDC) stock solution (with 1% pyridine added) was prepared at 0.25 M in water:ACN 1:1 v/v. The internal standard, 4-methylvaleric acid, was added. Samples were mixed with NPH and EDC stocks at a 1:1:1 volume ratio. The mixture was heated in a heating block at 60 °C for 30 min. Samples were then filtered through 0.22 μm filters and transferred into HPLC vials, which were stored at 4 °C before analysis.

An Agilent 6460 Triple Quad MS–MS was used to detect the derivatized butyrate. Both derivatized butyrate-NPH and 4-methylvaleric-NPH were detected in negative mode. Column: Thermo Scientific C18 4.6 × 50 mm, 1.8 μm particle size, at room temperature. Mobile phase A, water with 0.1% v/v formic acid. Mobile phase B, ACN with 0.1% v/v formic acid. Injection volume, 5.0 μl. Flow rate, 0.5 ml min^−1^. Gradient of solvent: 15% mobile phase B at 0.0 min; 100% mobile phase B at 3.5 min; 100% mobile phase B at 6.0 min; 15% mobile phase B at 6.5 min. The multiple sclerosis conditions were optimized using pure butyrate-NPH or 4-methylvaleric-NPH at 1 mM. The fragment voltage was set to 135 V, and the collision energy was 18 V. Multiple reaction monitoring (MRM) of 222 → 137 was assigned to butyrate, and MRM of 250 → 137 was assigned to 4-methylvaleric acid as the internal standard. The ratio between MRM of butyrate and 4-methylvaleric acid was used to quantify butyrate concentration. The final butyrate content in each organ was normalized by organ weight.

### SerBut administration in naive C57BL/6 mice

C57BL/6 mice, aged 8 weeks, were purchased from Charles River Laboratories and housed in the animal facility at the University of Chicago for 2 weeks before use. From day 0 to day 10, mice were administered twice-daily oral gavage of PBS, NaBut (15 mg, molar equivalent to SerBut) or SerBut (24 mg). On day 10, mice were killed. The lymph nodes (cervical and iliac, mesenteric and hock draining), spleen and lamina propria were collected and processed for flow cytometry analysis.

### CAIA model

BALB/c mice, aged 6 weeks, were purchased from the Jackson Laboratory and housed in the animal facility at the University of Chicago for 2 weeks before immunization. Mice were orally gavaged with PBS or SerBut (25 mg) once daily starting on day −14 or with PBS, NaBut (15 mg, molar equivalent to SerBut) or SerBut (24 mg) twice daily beginning on day 3 at the age of 8 weeks. CAIA was induced by passive immunization with an anti-collagen antibody cocktail (1 mg per mouse by intraperitoneal (i.p.) injections, Arthrogen-CIA 5-Clone Cocktail Kit, Chondrex) on day 0, followed by an i.p. injection of LPS (25 μg) on day 3. Mice cages were layered with soft (pine) bedding throughout the experiment. Arthritis severity was monitored daily after day 3 using the criteria for clinical scores established by Chondrex, as described previously^62^.

On day 12, the thickness of mouse fore- and hindpaws was measured to assess the swelling resulting from arthritis. On day 13, mice were killed, and the spleen and hock-draining LNs, including popliteal, axillary and brachial LNs, were collected for immunostaining, followed by flow cytometry analysis.

The paws were collected for histological analysis, as described previously^62^. Briefly, paws were fixed in 2% paraformaldehyde (Thermo Scientific), decalcified in Decalcifier II (Leica) and stored in 70% ethanol until paraffin embedding. Paraffin-embedded paws were sliced into 5-μm-thick sections and stained with haematoxylin and eosin or Masson’s trichrome. The images were captured using a CRi Pannoramic SCAN 40x or MIDI 20x whole slide scanner, or an Olympus VS200 slideview research slide scanner, and analysed using ImageJ and QuPath software.

### EAE model

C57BL/6 female mice (7–8 weeks old) were purchased from Charles River Laboratories and housed in the animal facility at the University of Chicago for 2 weeks before immunization. Female C57BL/6 mice, aged 10 weeks, were subcutaneously immunized at the dorsal flanks with an emulsion of MOG_35–__55_ in complete Freund’s adjuvant (MOG_35–__55_/CFA Emulsion, Hooke Laboratories) on day 0, followed by i.p. administration of pertussis toxin (140 ng) in PBS on both days 0 and 1. The development of EAE was monitored, and clinical scores were measured daily from day 7 to day 20. The criteria for clinical scores were according to the instructions from Hooke Laboratories and described previously^90^.

In the experiment from Fig. 5, mice were given drinking water containing 100 mM NaBut, l-Ser or SerBut from day −14 until the end of the study. On day 2 after EAE induction, PBS, NaBut (15 mg, molar equivalent to SerBut), l-Ser (12 mg, molar equivalent to SerBut) or SerBut (24 mg) was administered once daily by oral gavage. In the experiment from Fig. 6, mice were administered of PBS or SerBut (24 mg) twice daily by oral gavage from day 2 after EAE induction.

On day 21 or 22, mice were sacrificed. The spinal cords were collected and separated into three sections for immunofluorescence imaging, cytokine measurement through homogenization or immunostaining for flow cytometry analysis. Blood was collected through cardiac puncture, and the spleen, mLNs and SC-dLNs (including cervical LNs and iliac LNs) were collected. Single-cell suspensions were collected for immunostaining, followed by flow cytometry analysis. Major cytokines from the plasma and spinal cord after homogenization were analysed via LEGENDplex (BioLegend).

SJL/JCrHsd female mice (6 weeks old) were purchased from Envigo Laboratories and housed in the animal facility at the University of Chicago for 2 weeks before immunization. Female SJL/JCrHsd mice, aged 8 weeks, were subcutaneously immunized at the dorsal flanks with an emulsion of PLP_139__−__151_ in complete Freund’s adjuvant (PLP_139__−__151_ (naive)/CFA Emulsion, Hooke Laboratories) on day 0, followed by i.p. administration of pertussis toxin (100 ng) in PBS on both days 0 and 2. The development of relapsing–remitting EAE was monitored, and clinical scores were measured daily from day 7 to day 40. Clinical score was determined according to the instructions from Hooke Laboratories and described previously^90,91^. On day 19 after EAE induction, mice were assigned to treatment groups. Mice in each treatment group had the same average clinical score. From day 19 to endpoint, mice were administered twice-daily oral gavage of PBS or SerBut (24 mg). On day 40, mice were killed and tissues were processed for flow cytometry analysis, as described above.

### Immunofluorescence imaging of spinal cord sections

Thoracic and lumbar spines of EAE mice were collected. The tissues were fixed in 2% paraformaldehyde (Thermo Scientific) and then stored in 70% ethanol until paraffin embedding. Paraffin-embedded spinal cords were sliced into 5-μm-thick sections, as described previously^62,90^. The sections were deparaffinized through a series of washes in xylene, ethanol and double-distilled water. Spinal cord sections were immersed in each solution for 2 min per wash. Antigen retrieval was performed using 1× pH 6.0 citrate buffer at 50–55 °C for 45 min. Sections were blocked for 1 h at room temperature in PBS containing 0.3% Triton-X and 5% normal goat serum. Primary antibodies against CD45 (clone 30-F11, BioLegend) and MBP (clone ab40390, Abcam) were applied at 1:100 dilution in blocking buffer and incubated for 16 h at 4 °C. Sections were washed and then incubated with donkey anti-rat IgG (H + L) AF647 (A48272, Invitrogen) and donkey anti-rabbit IgG (H + L) AF488 (2340683, Jackson ImmunoResearch) secondary antibodies for 16 h at 4 °C. Following additional washes, sections were mounted using Fluoromount-G mounting medium and imaged with an Olympus IX83 spinning-disc confocal fluorescence microscope. Image processing was performed using ImageJ and QuPath software.

### Evaluation of immune responses to vaccination and safety profile of SerBut

Mice were orally gavaged with PBS, SerBut (twice daily, 24 mg per dose) or FTY720 (once daily, 0.02 mg per dose) starting on day −3 until the end of the experiment. On day 0, mice were immunized subcutaneously in the front hocks with 10 μg endotoxin-free OVA, 50 μg alum and 5 μg MPLA, as described previously^62^. Mice were bled on days 9 and 13, and plasma was analysed for anti-OVA total IgG antibodies using a mouse anti-OVA IgG antibody assay kit (Chondrex). On day 13, mice were killed, the hock-draining LNs and spleen were collected, and cells were isolated for immunostaining, followed by flow cytometry analysis. One million cells from each spleen were seeded in a 96-well plate and incubated with OVA at 100 μg ml^−1^ for a 3-day, ex vivo restimulation. The supernatant of cell culture was collected, and cytokines were measured using a LEGENDplex mouse T_H_ cytokine assay (BioLegend).

In addition, plasma samples collected on day 13 were analysed for various markers of organ toxicity using a biochemistry analyser (Alfa Wassermann Diagnostic Technologies) according to the manufacturer’s instructions. The panel included albumin, alanine aminotransferase, amylase, aspartate aminotransferase, blood urea nitrogen, calcium, creatine kinase, creatine, total bilirubin and total protein.

### Statistical analysis

Statistical analysis and plotting of data were performed using Prism 9.0 (GraphPad), as indicated in the figure legends. One-way analysis of variance (ANOVA) with Dunnett’s, Tukey’s or Kruskal–Wallis test (if not normally distributed) for multiple comparisons was used in Figs. 1d–h, 2b–h, 4b–g, 5b,d–n and 7b,c,e,g,i and Extended Data Fig. 4. Student’s *t*-test was used in Figs. 3b–d,h–m and 6b,d,f–o and Extended Data Figs. 1 and 2. Two-way ANOVA with Tukey’s or Bonferroni’s post-test was used in Extended Data Fig. 3. In Figs. 3b, 4b, 5b and 6b, the area under the curve values of clinical scores were compared using one-way ANOVA with Dunnett’s post-test or Student’s *t*-test. In Figs. 5c and 6c, the probability curve of EAE clinical scores remaining below 1.0 was compared between each two groups using the log-rank (Mantel–Cox) test. Data represent mean ± s.e.m.; *n* is stated in the figure legend.

### Reporting summary

Further information on research design is available in the Nature Portfolio Reporting Summary linked to this article.

### Supplementary information

Supplementary Information Supplementary Figs. 1–24.

Reporting Summary

### Source data

Source Data Figs. 1–7 and Extended Data Figs. 1–4 Source data.

Source Data Fig. 1b Unprocessed western blots.

Source Data Fig. 2b Unprocessed western blots.

## Data Availability

The data supporting the results in this study are available within the paper and its Supplementary Information. Source data are provided with this paper.
