# Supplementary material for: A serine-conjugated butyrate prodrug with high oral bioavailability suppresses autoimmune arthritis and neuroinflammation in mice
Source: Nat Biomed Eng. 2024 Apr 1;8(5):611–27. doi: 10.1038/s41551-024-01190-x (PMC11161413; doi:10.1038/s41551-024-01190-x)
Supplement: Supplementary file 1 — Supplementary Figs. 1–24. [file 41551_2024_1190_MOESM1_ESM.pdf]

nature biomedical engineering

Article https://doi.org/10.1038/s41551-024-01190-x

A serine-conjugated butyrate prodrug with high oral bioavailability suppresses autoimmune arthritis and neuro-inflammation in mice

In the format provided by the authors and unedited

Contents

Supplementary Fig. 1 | ^{1}H NMR spectrum of *O*-butyryl-*L*-serine (SerBut)

Supplementary Fig. 2 | SerBut suppressed BMDC activation.

Supplementary Fig. 3 | Flow cytometry gating strategy for the identification of MHCII^{+}, CD80^{+}, or CD86^{+} cells of BMDCs.

Supplementary Fig. 4 | Butyrate levels in plasma at 30 min after SerBut or NaBut oral administration to C57BL/6 mice.

Supplementary Fig. 5 | Histology images of mouse joints from paws stained with hematoxylin and eosin, from the experiment in Fig. 3.

Supplementary Fig. 6 | Histology images of mouse joints from paws stained with Masson's trichrome, from the experiment in Fig. 3.

Supplementary Fig. 7 | Measurement of the amount of drinking water consumed by healthy C57BL/6 mice.

Supplementary Fig. 8 | Flow cytometry gating strategy for the identification of PD-1^{+}, CTLA-4^{+}, or Foxp3^{+}CD25^{+} of CD4^{+} T cells, PD-1^{+} or CTLA-4^{+} of Foxp3^{+}CD25^{+}CD4^{+} Tregs, MOG tetramer-positive CD4^{+} or CD4^{+}RORγt^{+} T cells, and Foxp3^{+}CD25^{+}, PD-1^{+}, CTLA-4^{+} of MOG tetramer-positive CD4^{+} T cells.

Supplementary Fig. 9 | The gating strategy (**a**) and the percentage of Foxp3^{+} (**b**) or PD-1^{+} (**c**) of CD8^{+}CD4^{-} T cells in the spinal cord-draining lymph nodes (SC-dLNs, iliac and cervical LNs) measured by flow cytometry, from the experiment in Fig. 4.

Supplementary Fig. 10 | The percentage of co-stimulatory molecule (CD40^{+} and CD86^{+}) and MHCII^{+} cells of myeloid cells in the SC-dLNs from Fig. 5n.

Supplementary Fig. 11 | The percentage of CD11b^{+}Ly6C^{+}Ly6G^{-} cells in the spinal cord-draining LNs (**a**) or mesenteric LNs (**b**) from the experiment in Fig. 5.

Supplementary Fig. 12 | a. Flow cytometry gating strategy for CD11b^{+}CD11c^{-}, CD11b^{+}F4/80^{+}, CD11c^{+}, CD11b^{+}CD11c^{+}, CD11b^{+}Ly6C^{+}Ly6G^{-} cells **b**. Representative gating strategy for CD40^{+}, CD86^{+}, or MHCII^{+} cells of CD11b^{+}F4/80^{+} with FMO.

Supplementary Fig. 13 | Oral gavage of SerBut ameliorates EAE more effectively than SerBut in drinking water.

Supplementary Fig. 14 | Immunofluorescent images of spinal cord sections from mice treated with PBS or SerBut, taken from the experiment in Fig. 6.

Supplementary Fig. 15 | The percentage of Foxp3^{+}CD4^{+}, IL-17A^{+}CD4^{+}, and IFNγ^{+}CD4^{+} cells of live cells in the spinal cord, from the experiment in Fig. 6.

Supplementary Fig. 16 | The effect of SerBut on PD-1 expression of T cells, and PD-L1 expression on myeloid cells in mesenteric LNs from the therapeutic EAE experiment in Extended Data Fig. 2.

Supplementary Fig. 17 | Flow cytometry gating strategy for CD11b^{+}CD45^{low} spinal cord microglia in Extended Data Fig. 2c.

Supplementary Fig. 18 | Flow cytometry gating strategy for RORγt$^+$ FoxP3$^-$ CD4$^+$ T cells, FoxP3+ CD4$^+$ T cells, PD-1$^+$ CD4$^+$ T cells, PD-1$^+$ FoxP3$^+$ CD4$^+$ T cells, PD-L1$^+$ CD11c$^+$ CD8$^+$ cells, CD11b$^+$ CD11c$^+$ myeloid cells, PD-L1$^+$ CD11c$^+$ CD11b$^+$ cells, and PD-L1$^+$ CD11c$^+$ CD11b$^-$ cells.

Supplementary Fig. 19 | Flow cytometry gating strategy for CD19$^+$B220$^+$, B cells in Fig. 7d, e.

Supplementary Fig. 20 | The percentages of CD19$^+$B220$^+$ (**a**), CD3$^+$ (**b**), CD4$^+$ (**c**) of total live cells and Foxp3$^+$ of CD4$^+$ T cells (**d**) in the hock-draining LNs, from the experiment in Fig. 7.

Supplementary Fig. 21 | Serological toxicity analysis of mouse serum samples from the experiment in Fig. 7.

Supplementary Fig. 22 | Immunological effects of SerBut treatment in healthy C57BL/6 mice on the Tregs and Th17 cells in the ileum lamina propria from healthy mice treated with PBS, NaBut, or SerBut from Extended Data Fig. 4.

Supplementary Fig. 23 | The percentage of co-stimulatory molecule CD86$^+$ and MHCII$^+$ cells of myeloid cells in the mesenteric LNs from healthy mice treated with PBS, NaBut, or SerBut from Extended Data Fig. 4.

Supplementary Fig. 24 | Flow cytometry gating strategy for PD-1$^+$ CD4$^+$ T cells, CTLA-4$^+$ CD4$^+$ T cells, RORγt$^+$ FoxP3$^-$ CD4$^+$ T cells, Gata3$^+$ FoxP3$^-$ CD4$^+$ T cells, and Tbet$^+$ FoxP3$^-$ CD4$^+$ T cells in the spleen in Extended Data Fig. 4c.

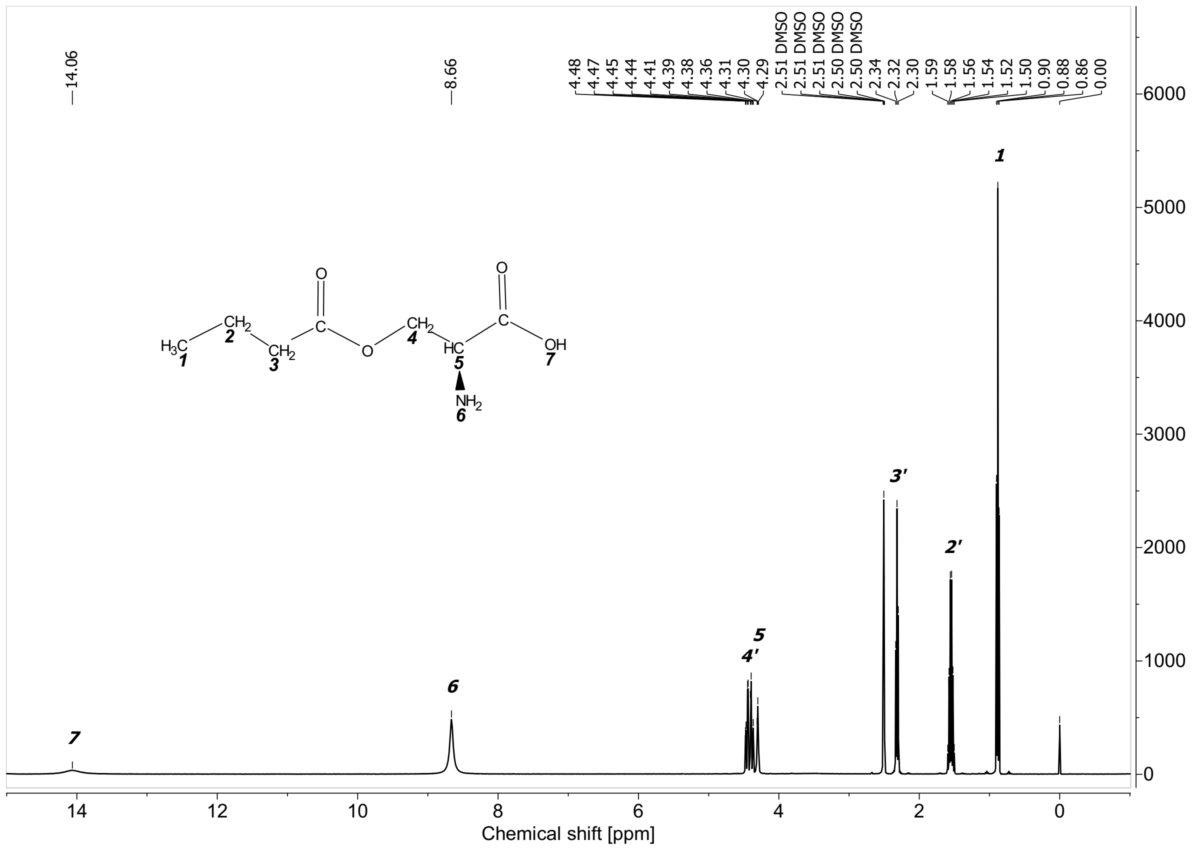

Supplementary Fig. 1 | ^{1}H NMR spectrum of *O*-butyryl-*L*-serine (SerBut) (500MHz, DMSO-d_6) [ppm]: 0.88 (3H, t), 1.55 (2H, m), 2.32 (2H, t), 4.30 (1H, t), 4.43 (2H, d), 8.66 (2H, s), 14.06 (1H, s).

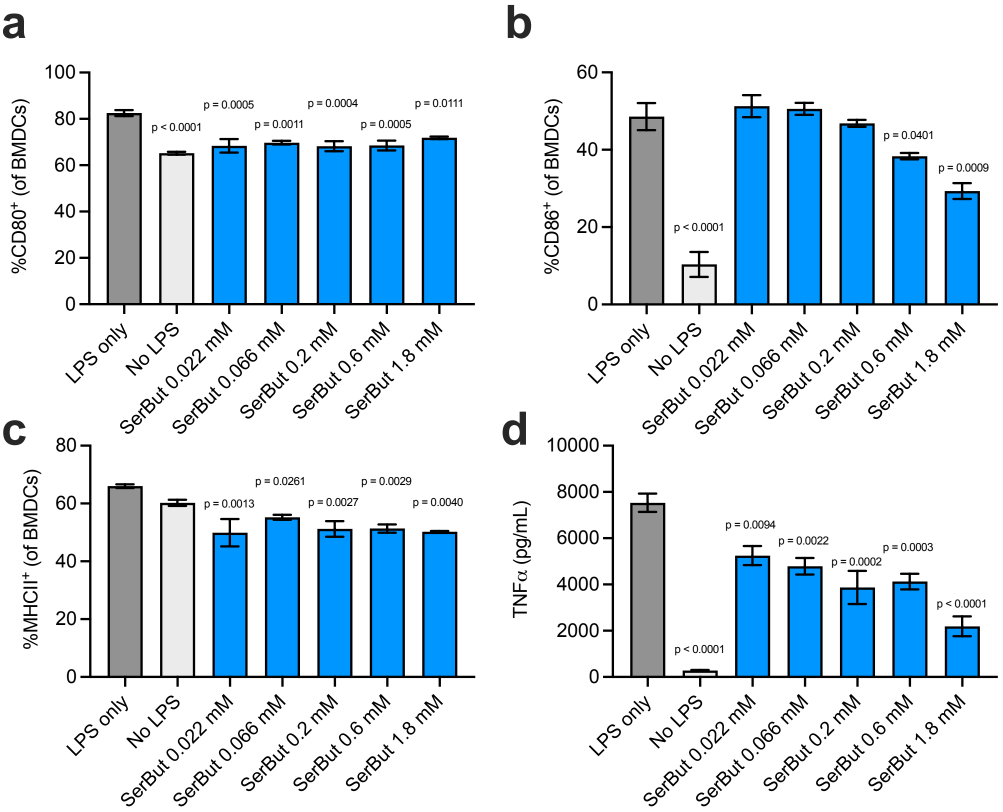

Supplementary Fig. 2 | SerBut suppressed BMDC activation. BMDCs incubated with SerBut at a series of concentrations for 24 hr, followed by LPS stimulation for 18 hr. **a-c**, Percentage of $CD80^+$, $CD86^+$, or $MHCII^+$ cells of BMDCs analyzed by flow cytometry. **d**. TNFα concentration in the cell culture supernatant of BMDCs. Data represent mean ± s.e.m. Statistical analyses were compared between LPS only group with no LPS or SerBut-treated group, performed using a one-way ANOVA with Dunnett's test. P value less than 0.05 were shown.

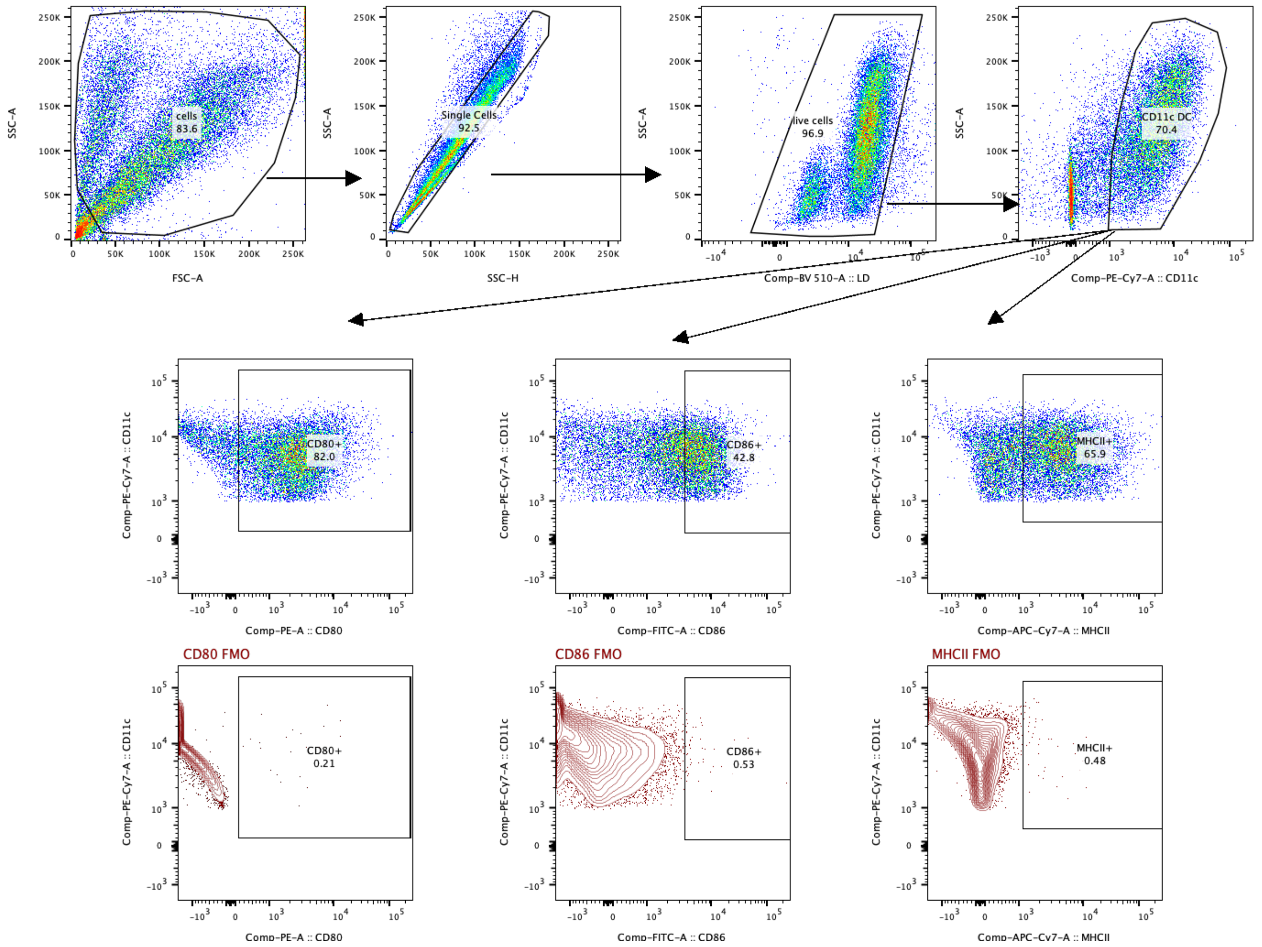

Supplementary Fig. 3 | Flow cytometry gating strategy for the identification of MHCII^{+}, CD80^{+}, or CD86^{+} cells of BMDCs. Representative FMOs (fluorescence minus one) were included for CD80, CD86, and MHCII gating strategy. This gating strategy was used in Fig.1d-f, and Supplementary Fig. 2.

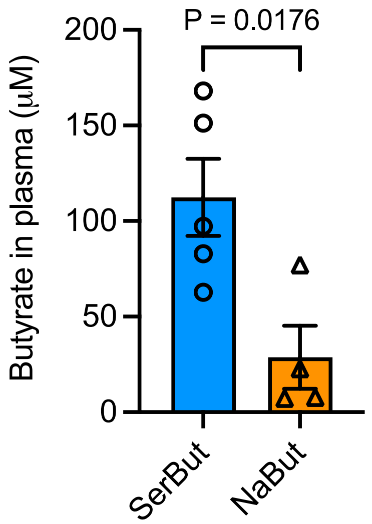

Supplementary Fig. 4 | Butyrate levels in plasma at 30 min after SerBut or NaBut oral administration to C57BL/6 mice. *n* = 5 mice per group. Data represent mean ± s.e.m. Statistical analyses were performed using Student's t-test.

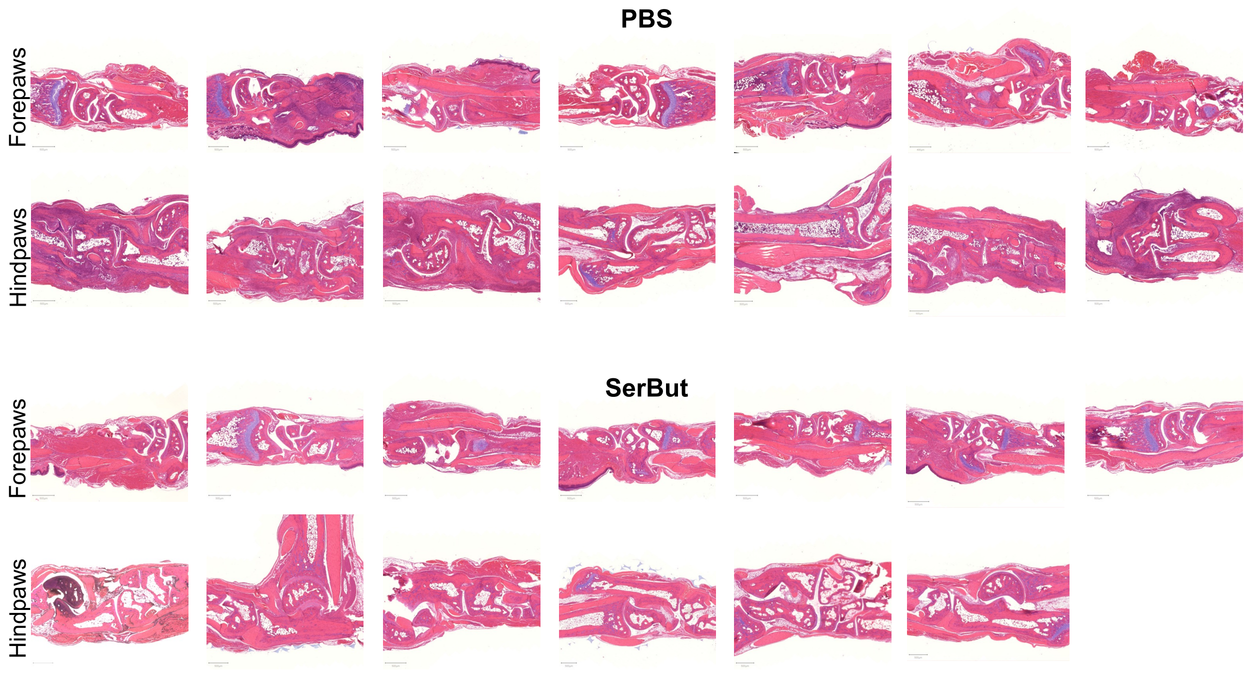

Supplementary Fig. 5 | Histology images of mouse joints from paws stained with hematoxylin and eosin, from the experiment in Fig. 3. (One hindpaw sample from SerBut group was lost during processing.) Scale bar, 500 µm.

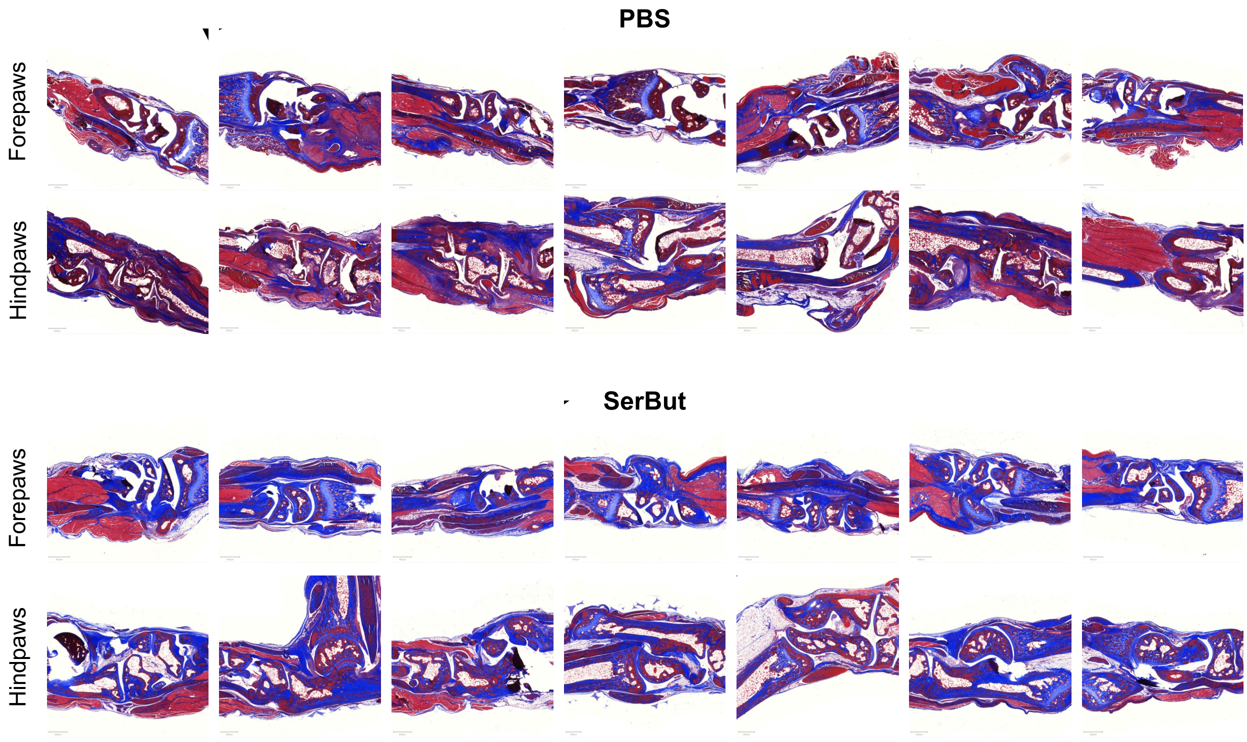

Supplementary Fig. 6 | Histology images of mouse joints from paws stained with Masson's trichrome, from the experiment in Fig. 3. Blue represents collagen staining.

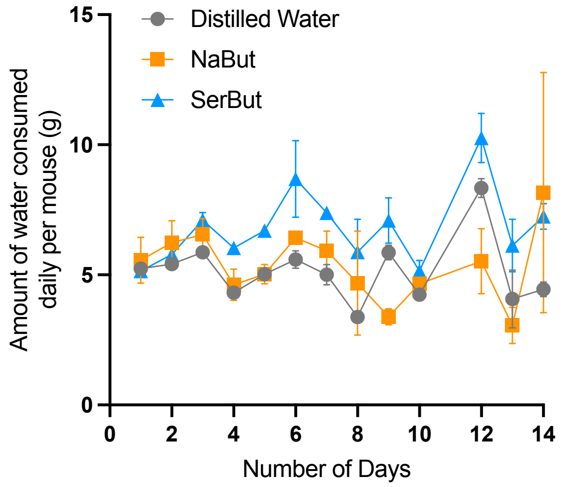

Supplementary Fig. 7 | Measurement of the amount of drinking water consumed by healthy C57BL/6 mice. Distilled water alone or supplemented with 100 mM NaBut or SerBut was administered to the mice over a 14-day period. Data represent mean ± s.e.m.

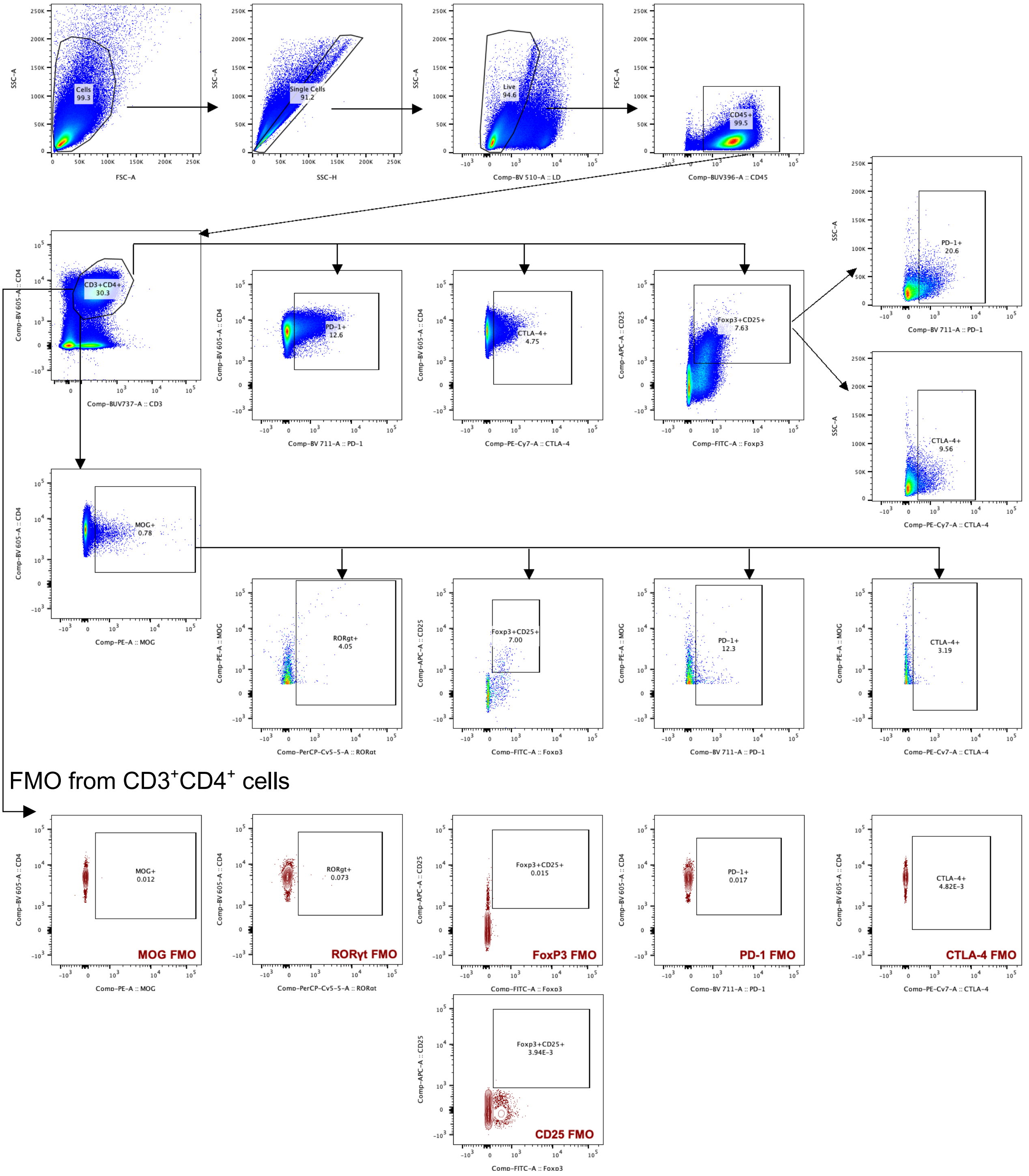

Supplementary Fig. 8 | Flow cytometry gating strategy for the identification of PD-1$^+$, CTLA-4$^+$, or Foxp3$^+$CD25$^+$ of CD4$^+$ T cells, PD-1$^+$ or CTLA-4$^+$ of Foxp3$^+$CD25$^+$CD4$^+$ Tregs, MOG tetramer-positive CD4$^+$ or CD4$^+$RORγt$^+$ T cells, and Foxp3$^+$CD25$^+$, PD-1$^+$, CTLA-4$^+$ of MOG tetramer-positive CD4$^+$ T cells. This gating strategy was used in Fig. 5, 6.

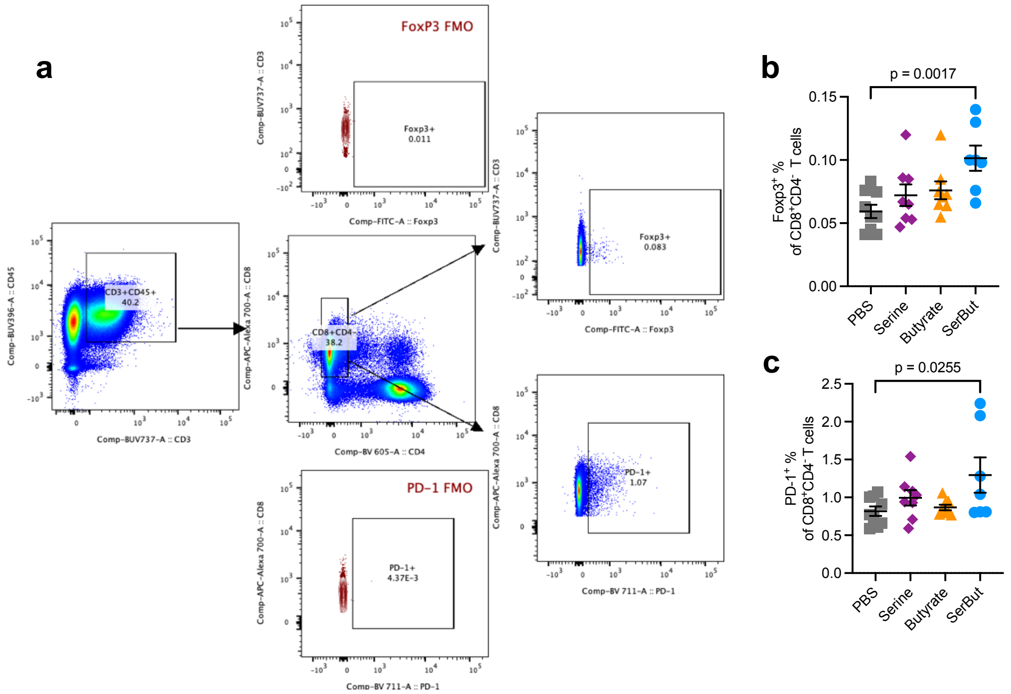

Supplementary Fig. 9 | The gating strategy (**a**) and the percentage of Foxp3$^+$ (**b**) or PD-1$^+$ (**c**) of CD8$^+$CD4$^-$ T cells in the spinal cord-draining lymph nodes (SC-dLNs, iliac and cervical LNs) measured by flow cytometry, from the experiment in Fig. 4. Data represent mean ± s.e.m. Statistical analyses were compared between PBS and each treatment group using one-way ANOVA with Dunnett's test. P values less than 0.05 were shown.

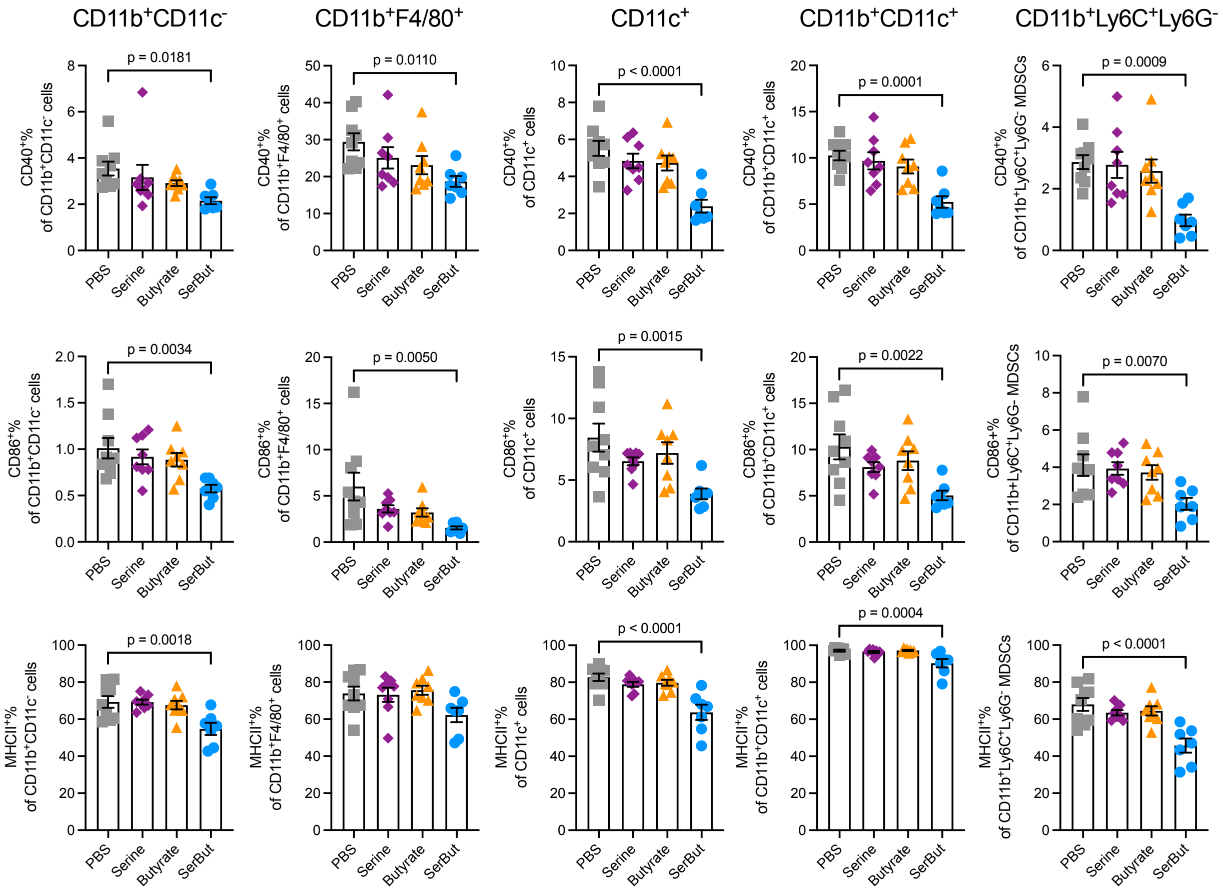

Supplementary Fig. 10 | The percentage of co-stimulatory molecule (CD40$^+$ and CD86$^+$) and MHCII$^+$ cells of myeloid cells in the SC-dLNs from Fig. 5n. Data represent mean ± s.e.m. Statistical analyses were compared between PBS and each treatment group using one-way ANOVA with Dunnett's test. P values less than 0.05 were shown.

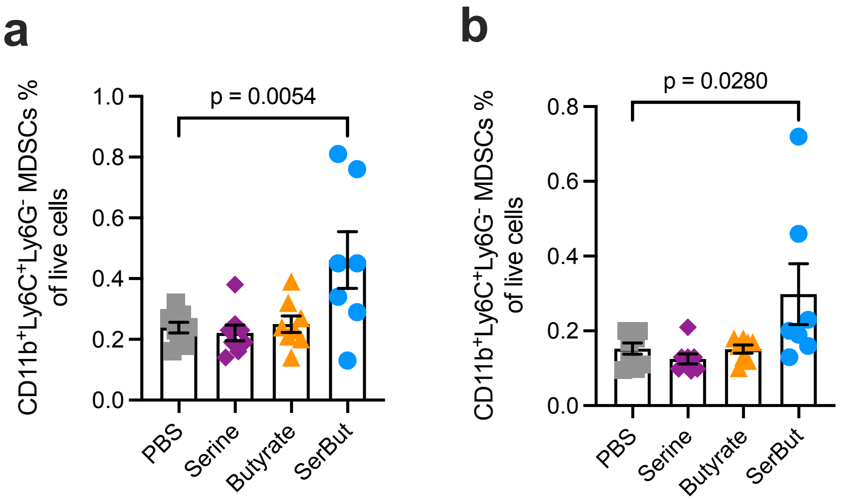

Supplementary Fig. 11 | The percentage of CD11b$^+$Ly6C$^+$Ly6G$^-$ cells in the spinal cord-draining LNs (**a**) or mesenteric LNs (**b**) from the experiment in Fig. 5. Data represent mean ± s.e.m. Statistical analyses were compared between PBS and each treatment group using one-way ANOVA with Dunnett's test. P values less than 0.05 were shown.

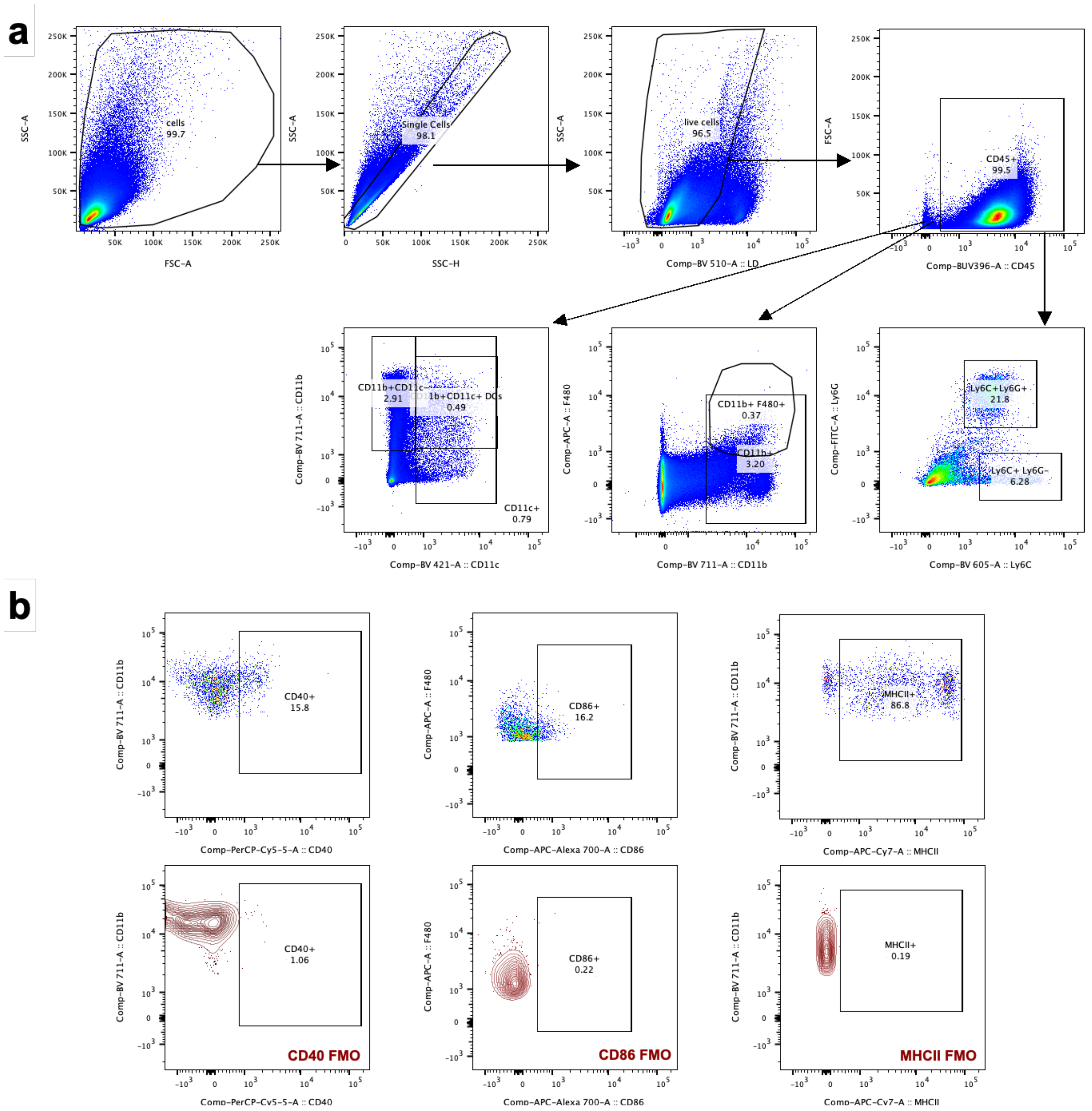

Supplementary Fig. 12 | a. Flow cytometry gating strategy for CD11b$^+$CD11c$^-$, CD11b$^+$F4/80$^+$, CD11c$^+$, CD11b$^+$CD11c$^+$, CD11b$^+$Ly6C$^+$Ly6G$^-$ cells **b**. Representative gating strategy for CD40$^+$, CD86$^+$, or MHCII$^+$ cells of CD11b$^+$F4/80$^+$ with FMO. This gating strategy was used in Fig. 5n, Supplementary Fig. 10 and Supplementary Fig. 11.

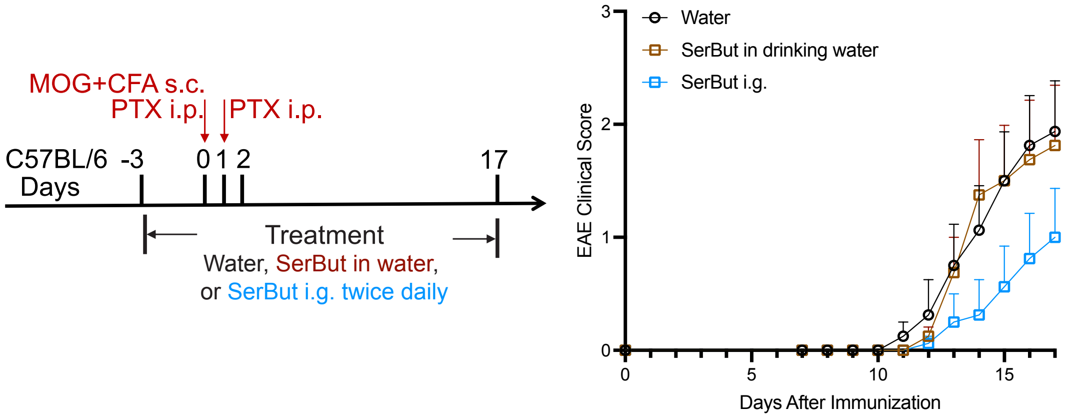

Supplementary Fig. 13 | Oral gavage of SerBut ameliorates EAE more effectively than SerBut in drinking water. EAE was induced in C57BL/6 using MOG_{35-55}/CFA with pertussis toxin. Mice were given regular drinking water, or drinking water containing 150 mM SerBut, or twice daily gavage of SerBut (24 mg per dose) from day -3 until the end of the study. Disease progression as indicated by the clinical score. *n* = 8 mice per group. Data represent mean ± s.e.m.

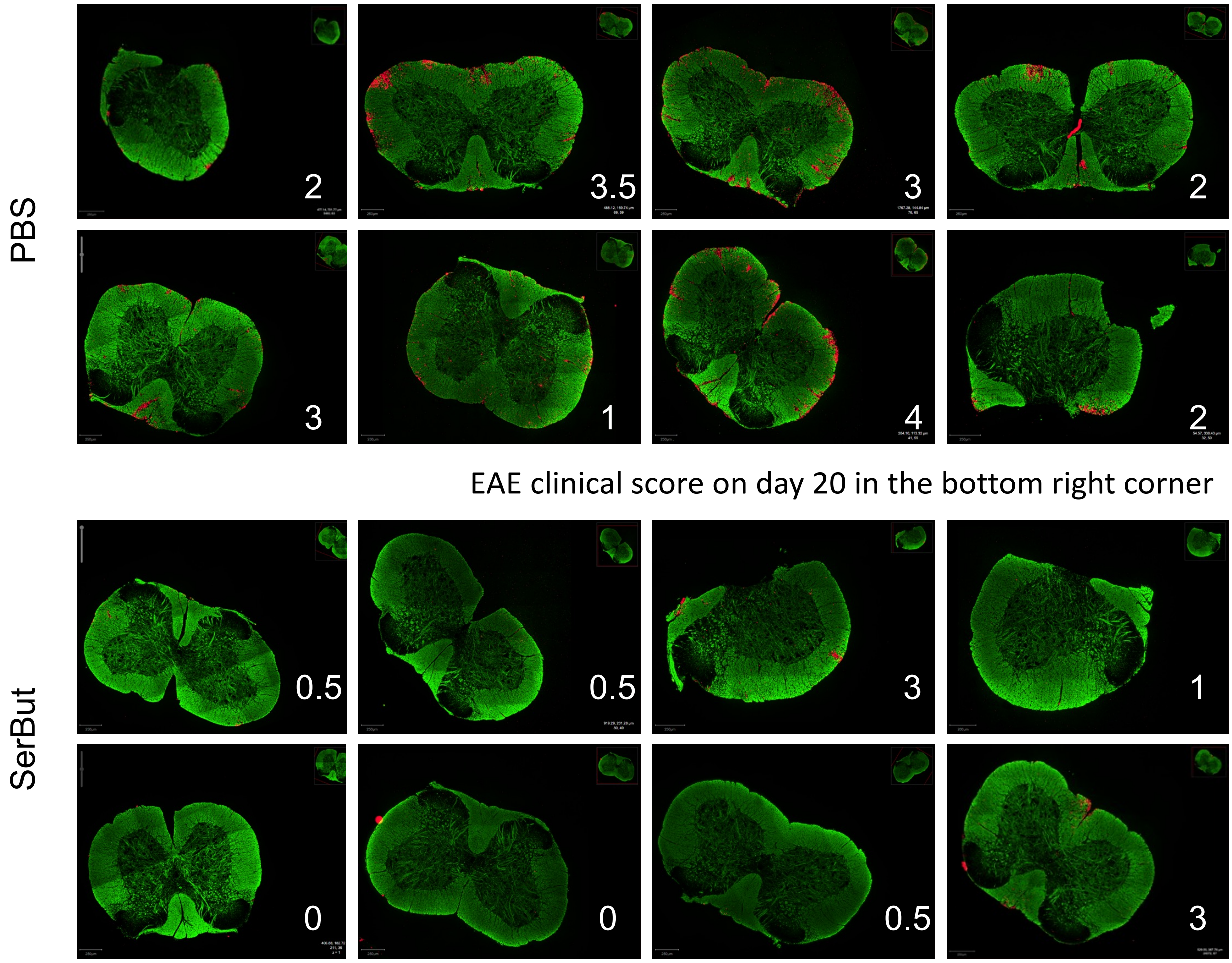

Supplementary Fig. 14 | Immunofluorescent images of spinal cord sections from mice treated with PBS or SerBut, taken from the experiment in Fig. 6. The EAE clinical score on day 20 is displayed in the bottom right corner of each image. Red: anti-CD45 staining; green: anti-myelin basic protein (MBP) staining.

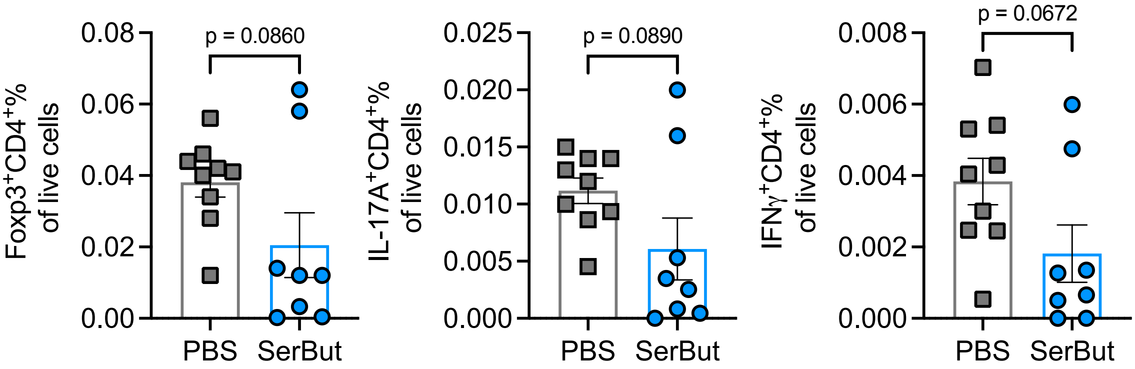

Supplementary Fig. 15 | The percentage of $Foxp3^{+}CD4^{+}$, IL-17$A^{+}CD4^{+}$, and IFN$\gamma^{+}CD4^{+}$ cells of live cells in the spinal cord, from the experiment in Fig. 6. Data represent mean ± s.e.m. Statistical analyses were performed using Student's t-test.

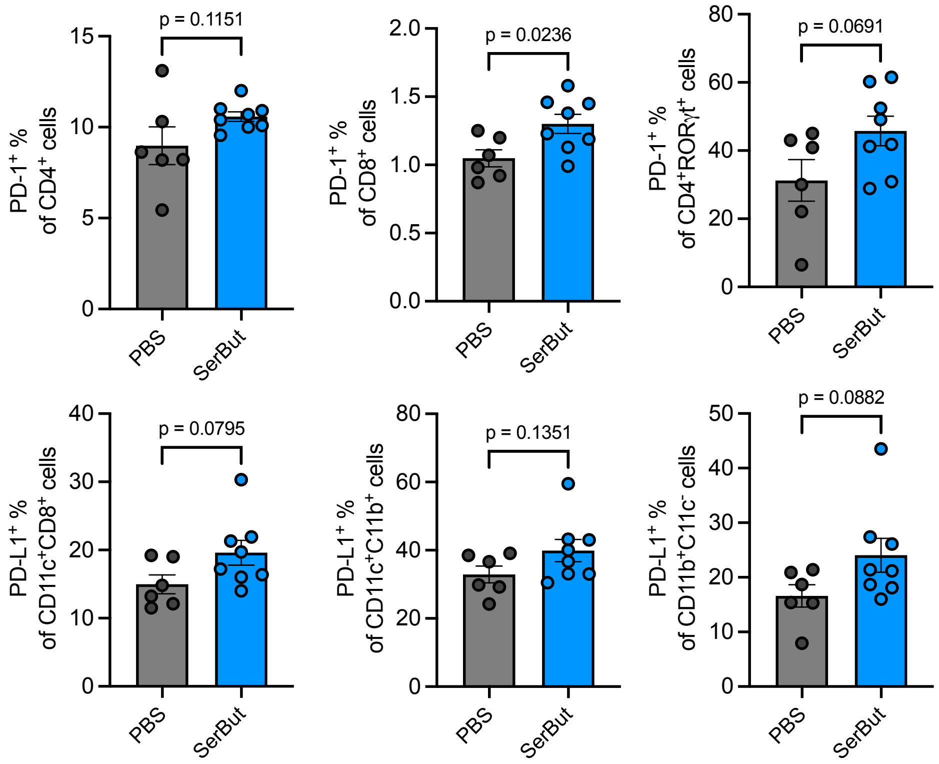

Supplementary Fig. 16 | The effect of SerBut on PD-1 expression of T cells, and PD-L1 expression on myeloid cells in mesenteric LNs from the therapeutic EAE experiment in Extended Data Fig. 2. Data represent mean ± s.e.m. Statistical analyses were performed using Student's t-test.

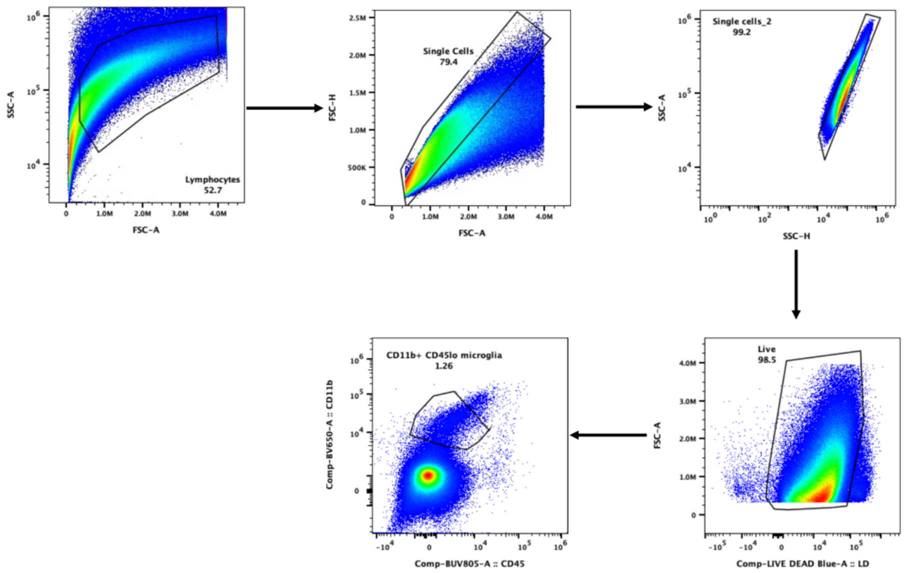

Supplementary Fig. 17 | Flow cytometry gating strategy for CD11b^{+}CD45^{low} spinal cord microglia in Extended Data Fig. 2c.

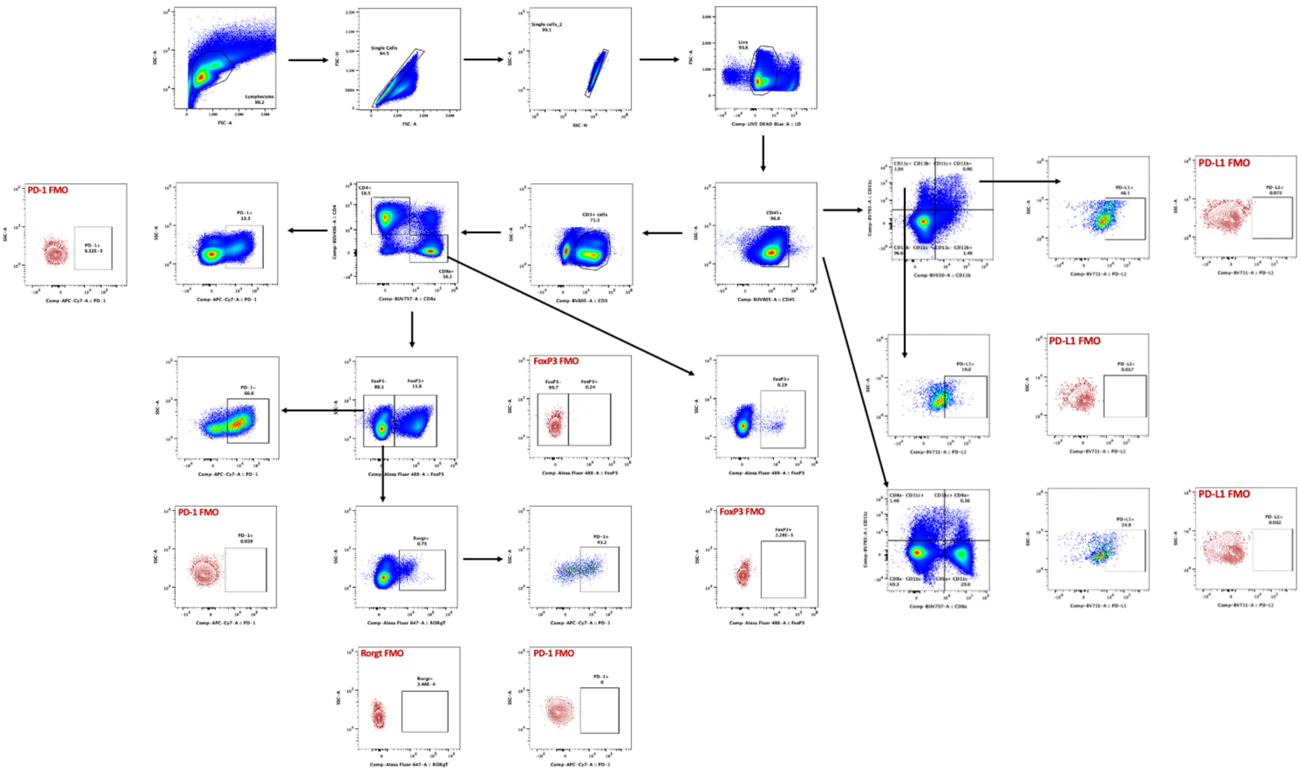

Supplementary Fig. 18 | Flow cytometry gating strategy for RORγt^{+} FoxP3^{-} CD4^{+} T cells, FoxP3+ CD4^{+} T cells, PD-1^{+} CD4^{+} T cells, PD-1^{+} FoxP3^{+} CD4^{+} T cells, PD-L1^{+} CD11c^{+} CD8^{+} cells, CD11b^{+} CD11c^{+} myeloid cells, PD-L1^{+} CD11c^{+} CD11b^{+} cells, and PD-L1^{+} CD11c^{+} CD11b^{-} cells. This gating strategy was used in Extended Data Fig. 2, and Supplementary Supplementary Fig. 16.

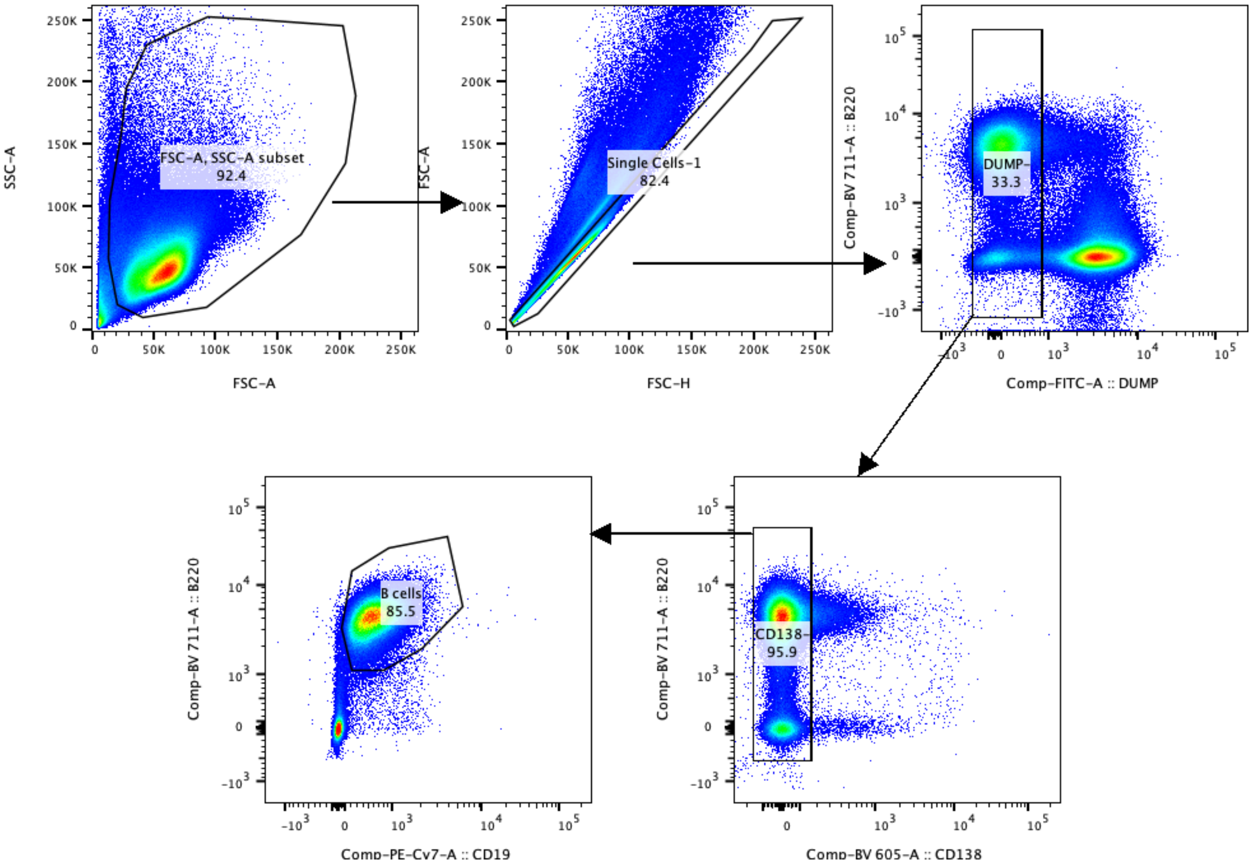

Supplementary Fig. 19 | Flow cytometry gating strategy for CD19^{+}B220^{+}, B cells in Fig. 7d, e. Dump gate to exclude cells stained with FITC antibody against F4/80, CD11c, Gr-1, CD4, and CD8.

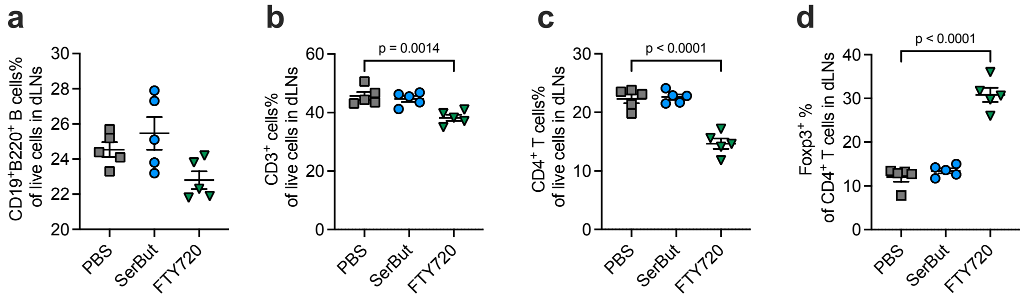

Supplementary Fig. 20 | The percentages of $CD19^{+}B220^{+}$ (**a**), $CD3^{+}$ (**b**), $CD4^{+}$ (**c**) of total live cells and $Foxp3^{+}$ of $CD4^{+}$ T cells (**d**) in the hock-draining LNs, from the experiment in Fig. 7. Data represent mean ± s.e.m. Statistical analyses were performed using one-way ANOVA with Dunnett's post hoc test. P values less than 0.05 were shown.

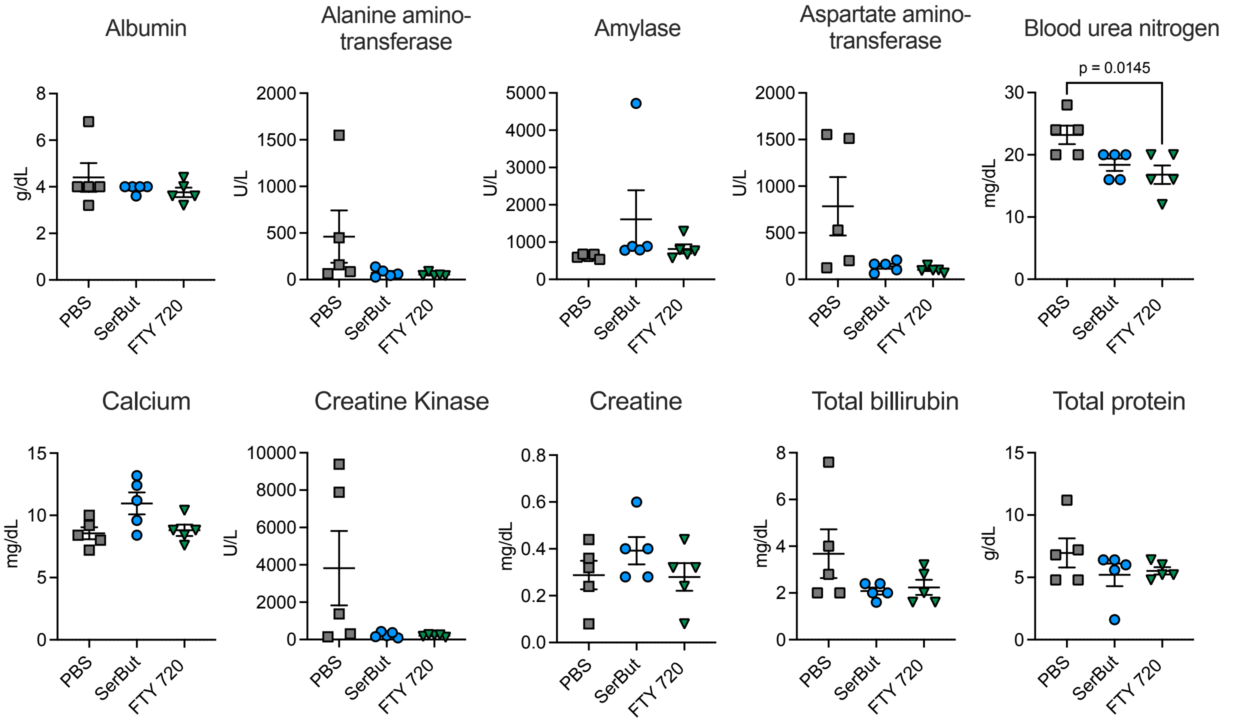

Supplementary Fig. 21 | Serological toxicity analysis of mouse serum samples from the experiment in Fig. 7. Data represent mean ± s.e.m. Statistical analyses were compared between every two groups using one-way ANOVA with Tukey's test. P values less than 0.05 were shown.

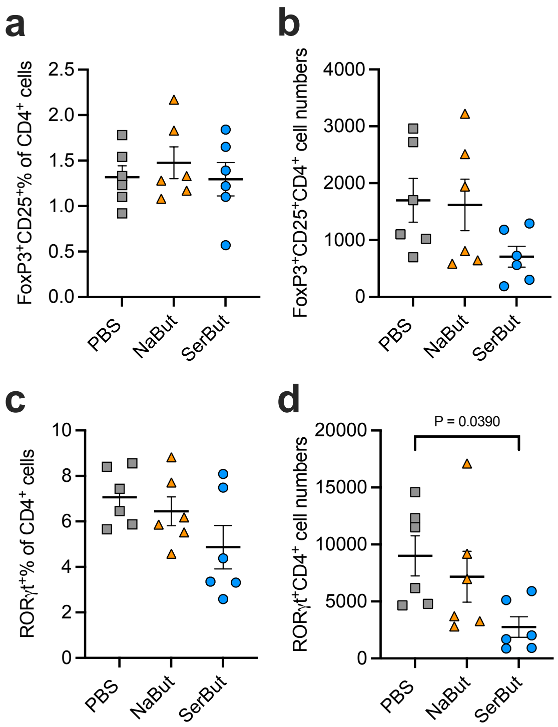

Supplementary Fig. 22 | Immunological effects of SerBut treatment in healthy C57BL/6 mice on the Tregs and Th17 cells in the ileum lamina propria from healthy mice treated with PBS, NaBut, or SerBut from Extended Data Fig. 4. **a, b.** The percentage of Foxp3$^+$CD25$^+$ of CD4$^+$ T cells and their cell numbers in the lamina propria. **c, d.** The percentage of RORγt$^+$ of CD4$^+$ T cells and their cell numbers in the laminar propria. Data represent mean ± s.e.m. Statistical analyses were compared between PBS and each treatment group using one-way ANOVA with Dunnett's test. P values less than 0.05 were shown.

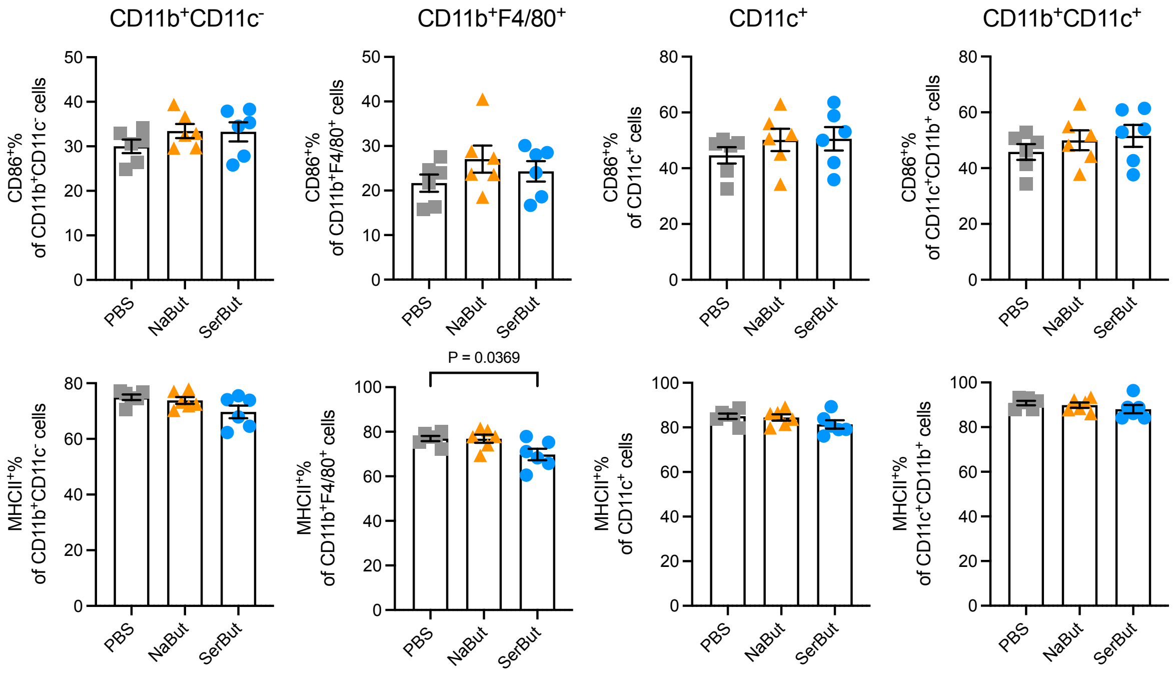

Supplementary Fig. 23 | The percentage of co-stimulatory molecule CD86$^+$ and MHCII$^+$ cells of myeloid cells in the mesenteric LNs from healthy mice treated with PBS, NaBut, or SerBut from Extended Data Fig. 4. Data represent mean ± s.e.m. Statistical analyses were compared between PBS and each treatment group using one-way ANOVA with Dunnett's test. P values less than 0.05 were shown.

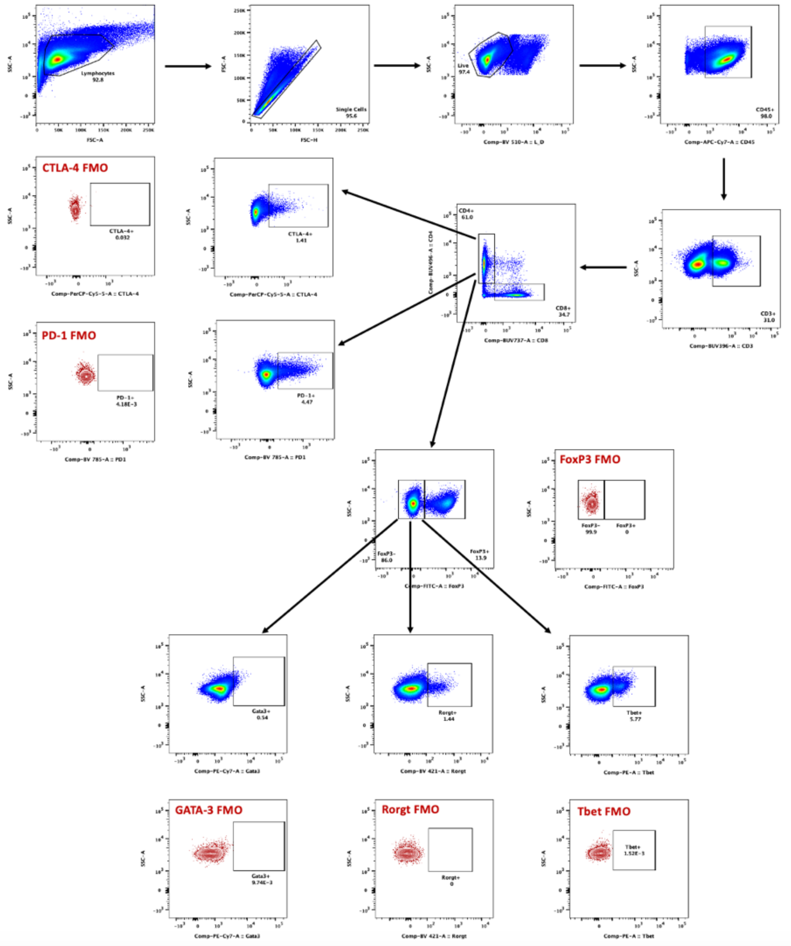

Supplementary Fig. 24 | Flow cytometry gating strategy for PD-1^{+} CD4^{+} T cells, CTLA-4^{+} CD4^{+} T cells, RORγt^{+} FoxP3^{-} CD4^{+} T cells, Gata3^{+} FoxP3^{-} CD4^{+} T cells, and Tbet^{+} FoxP3^{-} CD4^{+} T cells in the spleen in Extended Data Fig. 4c.
