# Supplementary material for: A serine-conjugated butyrate prodrug with high oral bioavailability suppresses autoimmune arthritis and neuroinflammation in mice
Source: Nat Biomed Eng. 2024 Apr 1;8(5):611–27. doi: 10.1038/s41551-024-01190-x (PMC11161413; doi:10.1038/s41551-024-01190-x)
Supplement: Supplementary file 2 — Reporting Summary [file 41551_2024_1190_MOESM2_ESM.pdf]

nature portfolio

Corresponding author(s): Jeffrey A. Hubbell, Shijie Cao

Last updated by author(s): Jan 8, 2024

Reporting Summary

Nature Portfolio wishes to improve the reproducibility of the work that we publish. This form provides structure for consistency and transparency in reporting. For further information on Nature Portfolio policies, see our Editorial Policies and the Editorial Policy Checklist.

Statistics

For all statistical analyses, confirm that the following items are present in the figure legend, table legend, main text, or Methods section.

n/a	Confirmed	
☐	☒	The exact sample size (*n*) for each experimental group/condition, given as a discrete number and unit of measurement
☐	☒	A statement on whether measurements were taken from distinct samples or whether the same sample was measured repeatedly
☐	☒	The statistical test(s) used AND whether they are one- or two-sided<br>*Only common tests should be described solely by name; describe more complex techniques in the Methods section.*
☒	☐	A description of all covariates tested
☐	☒	A description of any assumptions or corrections, such as tests of normality and adjustment for multiple comparisons
☐	☒	A full description of the statistical parameters including central tendency (e.g. means) or other basic estimates (e.g. regression coefficient) AND variation (e.g. standard deviation) or associated estimates of uncertainty (e.g. confidence intervals)
☐	☒	For null hypothesis testing, the test statistic (e.g. *F*, *t*, *r*) with confidence intervals, effect sizes, degrees of freedom and *P* value noted<br>*Give P values as exact values whenever suitable.*
☒	☐	For Bayesian analysis, information on the choice of priors and Markov chain Monte Carlo settings
☒	☐	For hierarchical and complex designs, identification of the appropriate level for tests and full reporting of outcomes
☒	☐	Estimates of effect sizes (e.g. Cohen's *d*, Pearson's *r*), indicating how they were calculated

Our web collection on statistics for biologists contains articles on many of the points above.

Software and code

Policy information about availability of computer code

Data collection	Bruker TopSpin (3.x) was used to collect NMR data. LSR Fortessa flow cytometer (BD Biosciences) was used to collect flow cytometry data.
Data analysis	ImageJ and QuPath software were used to analyze immunofluorescance images. PRISM software (GraphPad v9) was used to plot data and perform statistical analysis. FlowJo (v10.9.0) was used to analyze flow cytometry data. Microsoft excel was used to carry out simple operations.

For manuscripts utilizing custom algorithms or software that are central to the research but not yet described in published literature, software must be made available to editors and reviewers. We strongly encourage code deposition in a community repository (e.g. GitHub). See the Nature Portfolio guidelines for submitting code & software for further information.

Data

Policy information about availability of data

All manuscripts must include a data availability statement. This statement should provide the following information, where applicable:

- Accession codes, unique identifiers, or web links for publicly available datasets
- A description of any restrictions on data availability
- For clinical datasets or third party data, please ensure that the statement adheres to our policy

The main data supporting the results in this study are available within the paper and its Supplementary Information. Additional raw and processed datasets are available from the corresponding author upon reasonable request.

Research involving human participants, their data, or biological material

Policy information about studies with human participants or human data. See also policy information about sex, gender (identity/presentation), and sexual orientation and race, ethnicity and racism.

Reporting on sex and gender	No human participants were involved in this work.
Reporting on race, ethnicity, or other socially relevant groupings	No human participants were involved in this work.
Population characteristics	No human participants were involved in this work.
Recruitment	No human participants were involved in this work.
Ethics oversight	No human participants were involved in this work.

Note that full information on the approval of the study protocol must also be provided in the manuscript.

Field-specific reporting

Please select the one below that is the best fit for your research. If you are not sure, read the appropriate sections before making your selection.

☒ Life sciences ☐ Behavioural & social sciences ☐ Ecological, evolutionary & environmental sciences

For a reference copy of the document with all sections, see nature.com/documents/nr-reporting-summary-flat.pdf

Life sciences study design

All studies must disclose on these points even when the disclosure is negative.

Sample size	Sample size was determined using the results obtained from previous and preliminary studies. See figure legends for details on n for each display figure. For example we referred to the paper doi: 10.1038/s41551-020-00627-3, which has reported similar experimental procedures.
Data exclusions	No data was excluded from the analysis.
Replication	Experiments were repeated at least once, unless otherwise stated in the figure legend. Replicates were reproducible.
Randomization	The mice were randomized.
Blinding	EAE and CAIA clinical scores and weights were collected blindly. The person assessing clinical scores for RA and EAE experiments was separate from the person administering treatment and was blinded to the treatment group. The person performing fluorescent imaging and histology analysis was also blinded to treatment groups.

Reporting for specific materials, systems and methods

We require information from authors about some types of materials, experimental systems and methods used in many studies. Here, indicate whether each material, system or method listed is relevant to your study. If you are not sure if a list item applies to your research, read the appropriate section before selecting a response.

Materials & experimental systems

n/a	Involved in the study	
☐	☒	Antibodies
☒	☐	Eukaryotic cell lines
☒	☐	Palaeontology and archaeology
☐	☒	Animals and other organisms
☒	☐	Clinical data
☒	☐	Dual use research of concern
☒	☐	Plants

Methods

n/a	Involved in the study	
☒	☐	ChIP-seq
☐	☒	Flow cytometry
☒	☐	MRI-based neuroimaging

Antibodies

Antibodies used	For flow cytometry in the RA and EAE models, the following anti-mouse antibodies were used: INOS (BUV737, Cat#367-5920-82, Invitrogen), CD11c (BV421, Cat#562782, BD Biosciences), CD11c (BV785, Cat#563735, Biolegend), CD11b (BV650, Cat#563402, BD

Biosciences), Ly6C (BV605, Cat#128036, BioLegend), CD11b (BV711, Cat#101242, BioLegend), Ly6G (AF488, Cat#127626, BioLegend), CD40 (PerCP/Cy5.5, Cat#124624, BioLegend), CD40 (BUV615, Cat#751646, BD Biosciences), CD206 (PE, Cat#141706, BioLegend), CD206 (AF700, Cat#141734, Biolegend), Arginase 1 (PE-Cy7, Cat#25-3697-82, Invitrogen), F4/80 (APC, Cat#123116, BioLegend), F4/80 (PE, Cat#565410, BD Biosciences), CD86 (AF700, Cat#105024, BioLegend), CD86 (BUV395, Cat#564199, BD Biosciences), I- A/I-E (APC/Cy7, Cat#107628, BioLegend), I-A/I-E (BV421, Cat#107632, Biolegend), CD19 (BUV396, Cat#563557, BD Biosciences), CD3 (BUV737, Cat#741788, BD Biosciences), CD3 (BV605, Cat#100351, BioLegend), CD3 (APC-Fire750, Cat#100362, Biolegend), CD4 (BV605, Cat#100548, BioLegend), CD4 (BV711, Cat#100550, BioLegend), CD4 (BUV496, Cat#612952, BD Biosciences), CD4 (AF647, Cat#553051, BD Biosciences), PD-1 (BV711, Cat#135231, BioLegend), PD-1 (APC-Cy7, Cat#135223, BioLegend), PD-L1 (BV711, Cat#563369, BD Biosciences), Foxp3 (AF488, Cat#53-5773-82, Invitrogen), RORγt (PerCP/Cy5.5, Cat#562683, BD Biosciences), RORγt (APC, Cat#562682, BD Biosciences), RORγt (BV421, Cat#562894, BD Biosciences), CD5 (PE, Cat#100607, BioLegend), CTLA-4 (PE-Cy7, Cat#25-1522-80, Invitrogen), CTLA-4 (PE-Cy7, Cat#106314, Biolegend), CD25 (APC, Cat#162105, BioLegend), CD25 (PerCP/Cy5.5, Cat#561112, BD Biosciences), CD25 (BV650, Cat#10238, Biolegend), CD8 (AF700, Cat#100730, BioLegend), CD8 (BUV737, Cat#612759, BD Biosciences), IL-10 (APC/Cy7, Cat#505036, BioLegend), CD45 (BUV395, Cat#564279, BD Biosciences), CD45 (V450, Cat#560501, BD Biosciences), and CD45 (BUV805, Cat#748370, BD Biosciences). The I-A(b) mouse MOG 38-49 GWYRSPFSRVVH (MOG tetramer, PE) was obtained from NIH Tetramer Core Facility.

For flow cytometry in the mouse BMDC activation assay the following anti-mouse antibodies were used (all in 1:200 dilution): live/dead stain (Cat#L34957, Invitrogen), CD11c (PE-Cy7, Cat#558079, BD Biosciences), MHCII (APC-Cy7, Cat#107628, BioLegend), CD80 (PE, Cat#104708, BioLegend), CD86 (FITC, Cat#MA1-10300, Invitrogen).

For immunofluorescence staining of spinal cord sections, the following antibodies were used: Primary antibodies against CD45 (clone 30-F11, BioLegend) and MBP (clone ab40390, Abcam), and secondary antibodies donkey anti-rat IgG (H+L) AF647 (A48272, Invitrogen) and donkey anti-rabbit IgG (H+L) AF488 (2340683, Jackson ImmunoResearch).

Validation

INOS (BUV737, Cat#367-5920-82, Invitrogen): validated for mouse (QC testing) for flow cytometry (routinely tested)
CD11c (BV421, Cat#562782, BD Biosciences): validated for mouse (QC testing) for flow cytometry (routinely tested)
CD11c (BV785, Cat#563735, Biolegend): validated for mouse (QC testing) for flow cytometry (routinely tested)
Ly6C (BV605, Cat#128036, BioLegend): validated for mouse (QC testing) for flow cytometry (routinely tested)
CD11b (BV711, Cat#101242, BioLegend): validated for mouse (QC testing) for flow cytometry (routinely tested)
CD11b (BV650, Cat#563402, BD Biosciences): validated for mouse (QC testing) for flow cytometry (routinely tested)
Ly6G (AF488, Cat#127626, BioLegend): validated for mouse (QC testing) for flow cytometry (routinely tested)
CD40 (PerCP/Cy5.5, Cat#124624, BioLegend): validated for mouse (QC testing) for flow cytometry (routinely tested)
CD40 (BUV615, Cat#751646, BD Biosciences): validated for mouse (QC testing) for flow cytometry (routinely tested)
CD206 (PE, Cat#141706, BioLegend): validated for mouse (QC testing) for flow cytometry (routinely tested)
CD206 (AF700, Cat#141734, Biolegend): validated for mouse (QC testing) for flow cytometry (routinely tested)
Arginase 1 (PE-Cy7, Cat#25-3697-82, Invitrogen): validated for mouse (QC testing) for flow cytometry (routinely tested)
F4/80 (APC, Cat#123116, BioLegend): validated for mouse (QC testing) for flow cytometry (routinely tested)
F4/80 (PE, Cat#565410, BD Biosciences): validated for mouse (QC testing) for flow cytometry (routinely tested)
CD86 (AF700, Cat#105024, BioLegend): validated for mouse (QC testing) for flow cytometry (routinely tested)
I- A/I-E (APC/Cy7, Cat#107628, BioLegend): validated for mouse (QC testing) for flow cytometry (routinely tested)
I-A/I-E (BV421, Cat#107632, Biolegend): validated for mouse (QC testing) for flow cytometry (routinely tested)
CD19 (BUV396, Cat#563557, BD Biosciences): validated for mouse (QC testing) for flow cytometry (routinely tested)
CD3 (BUV737, Cat#741788, BD Biosciences): validated for mouse (QC testing) for flow cytometry (routinely tested)
CD3 (BV605, Cat#100351, BioLegend): validated for mouse (QC testing) for flow cytometry (routinely tested)
CD3 (APC-Fire750, Cat#100362, Biolegend): validated for mouse (QC testing) for flow cytometry (routinely tested)
CD4 (BV605, Cat#100548, BioLegend): validated for mouse (QC testing) for flow cytometry (routinely tested)
CD4 (BV711, Cat#100550, BioLegend): validated for mouse (QC testing) for flow cytometry (routinely tested)
CD4 (AF647, Cat#553051, BD Biosciences): validated for mouse (QC testing) for flow cytometry (routinely tested)
CD4 (BUV496, Cat#612952, BD Biosciences): validated for mouse (QC testing) for flow cytometry (routinely tested)
PD-1 (BV711, Cat#135231, BioLegend): validated for mouse (QC testing) for flow cytometry (routinely tested)
PD-1 (APC-Cy7, Cat#135223, BioLegend): validated for mouse (QC testing) for flow cytometry (routinely tested)
PD-L1 (BV711, Cat#563369, BD Biosciences): validated for mouse (QC testing) for flow cytometry (routinely tested)
Foxp3 (AF488, Cat#53-5773-82, Invitrogen): validated for mouse (QC testing) for flow cytometry (routinely tested)
RORγt (PerCP/Cy5.5, Cat#562683, BD Biosciences): validated for mouse (QC testing) for flow cytometry (routinely tested)
RORγt (APC, Cat#562682, BD Biosciences): validated for mouse (QC testing) for flow cytometry (routinely tested)
RORγt (BV421, Cat#562894, BD Biosciences): validated for mouse (QC testing) for flow cytometry (routinely tested)
CD5 (PE, Cat#100607, BioLegend): validated for mouse (QC testing) for flow cytometry (routinely tested)
CTLA-4 (PE-Cy7, Cat#25-1522-80, Invitrogen): validated for mouse (QC testing) for flow cytometry (routinely tested)
CTLA-4 (PE-Cy7, Cat#106314, Biolegend): validated for mouse (QC testing) for flow cytometry (routinely tested)
CD25 (APC, Cat#162105, BioLegend): validated for mouse (QC testing) for flow cytometry (routinely tested)
CD25 (PerCP/Cy5.5, Cat#561112, BD Biosciences): validated for mouse (QC testing) for flow cytometry (routinely tested)
CD25 (BV650, Cat#10238, Biolegend): validated for mouse (QC testing) for flow cytometry (routinely tested)
CD8 (BUV737, Cat#612759, BD Biosciences): validated for mouse (QC testing) for flow cytometry (routinely tested)
CD8 (AF700, Cat#100730, BioLegend): validated for mouse (QC testing) for flow cytometry (routinely tested)
IL-10 (APC/Cy7, Cat#505036, BioLegend): validated for mouse (QC testing) for flow cytometry (routinely tested)
CD45 (BUV395, Cat#564279, BD Biosciences): validated for mouse (QC testing) for flow cytometry (routinely tested)
CD45 (V450, Cat#560501, BD Biosciences): validated for mouse (QC testing) for flow cytometry (routinely tested)
CD11c (PE-Cy7, Cat#558079, BD Biosciences): validated for mouse (QC testing) for flow cytometry (routinely tested)
MHCII (APC-Cy7, Cat#107628, BioLegend): validated for mouse (QC testing) for flow cytometry (routinely tested)
CD80 (PE, Cat#104708, BioLegend): validated for mouse (QC testing) for flow cytometry (routinely tested)
CD86 (FITC, Cat#MA1-10300, Invitrogen): validated for mouse (QC testing) for flow cytometry (routinely tested)
CD86 (BUV395, Cat#564199, BD Biosciences): validated for mouse (QC testing) for flow cytometry (routinely tested)
CD45 (BUV805, Cat#748370, BD Biosciences): validated for mouse (QC testing) for flow cytometry (routinely tested)
CD45 (clone 30-F11, BioLegend): validated for mouse (QC testing) for flow cytometry and reported for immunohistochemistry
MBP (clone ab40390, Abcam): validated for mouse and rat (QC testing) for western blot and immunocytochemistry (routinely tested).
donkey anti-rat IgG (H+L) AF647 (A48272, Invitrogen): validated for rat (QC testing) for western blot and immunocytochemistry.

donkey anti-rabbit IgG (H+L) AF488 (2340683, Jackson ImmunoResearch): validated for rat (QC testing) for western blot and immunocytochemistry

Animals and other research organisms

Policy information about studies involving animals; ARRIVE guidelines recommended for reporting animal research, and Sex and Gender in Research

Laboratory animals	For MOG-EAE experiments, 8-week-old female C57BL/6 mice were purchased from Charles River Laboratories, and housed for two weeks at the University of Chicago animal facility prior to starting the experiments. For PLP-EAE experiment, 6-week old female SJL/JCrHsd mice were purchased from Envigo, and housed for two weeks at the University of Chicago animal facility prior to starting the experiment. For CAIA arthritis model, 6-week-old female BALB/C mice were purchased from the Jackson Laboratory and housed for two weeks at the University of Chicago animal facility prior to starting the experiments. For the vaccine study 8-week-old female C57BL/6 mice were purchased from Charles River Laboratories, and housed for 2 weeks at the University of Chicago animal facility prior to use.
Wild animals	This study did not involve wild animals.
Reporting on sex	These experiments were performed on female mice.
Field-collected samples	This study did not involve field collection of samples.
Ethics oversight	All experiments were approved by the University of Chicago's Institutional Animal Care and Use Committee (IACUC).

Note that full information on the approval of the study protocol must also be provided in the manuscript.

Flow Cytometry

Plots

Confirm that:

☒ The axis labels state the marker and fluorochrome used (e.g. CD4-FITC).

☒ The axis scales are clearly visible. Include numbers along axes only for bottom left plot of group (a 'group' is an analysis of identical markers).

☒ All plots are contour plots with outliers or pseudocolor plots.

☒ A numerical value for number of cells or percentage (with statistics) is provided.

Methodology

Sample preparation	Briefly, the spinal cord was collected and then digested in 5 mL DMEM (Gibco) with 2 mg/mL Collagenase D (Sigma), 20 ug/mL DNAse I (Worthington Biochemical) and 1.2 mM CaCl2 for 60 min at 37 C on a shaker for 30 min. After quenching the media with 5 mM EDTA (Gibco), single-cell suspensions were prepared using a 70-um cell strainer (Fisher). Spinal cord, LN and spleen single cell samples were prepared by mashing tissue on the a 70-um cell strainer (Fisher). For spleen samples, red blood cells were lysed with 1 mL ACK lysing buffer (Gibco) for 90 sec and neutralized with 10 mL DMEM media with 5% FBS.
Instrument	Sample collection was performed on an LSR Fortessa flow cytometer (BD Biosciences).
Software	FlowJo (v10.9.0) was used to analyze flow cytometry data.
Cell population abundance	Cells were not sorted for any of the experiments.
Gating strategy	Representative gating strategies are shown in figure S3, S8, S9, S12, S17, S18, S19, and S24.

☒ Tick this box to confirm that a figure exemplifying the gating strategy is provided in the Supplementary Information.
