# Supplementary figures and images for: A serine-conjugated butyrate prodrug with high oral bioavailability suppresses autoimmune arthritis and neuroinflammation in mice
Source: Nat Biomed Eng. 2024 Apr 1;8(5):611–27. doi: 10.1038/s41551-024-01190-x (PMC11161413; doi:10.1038/s41551-024-01190-x)

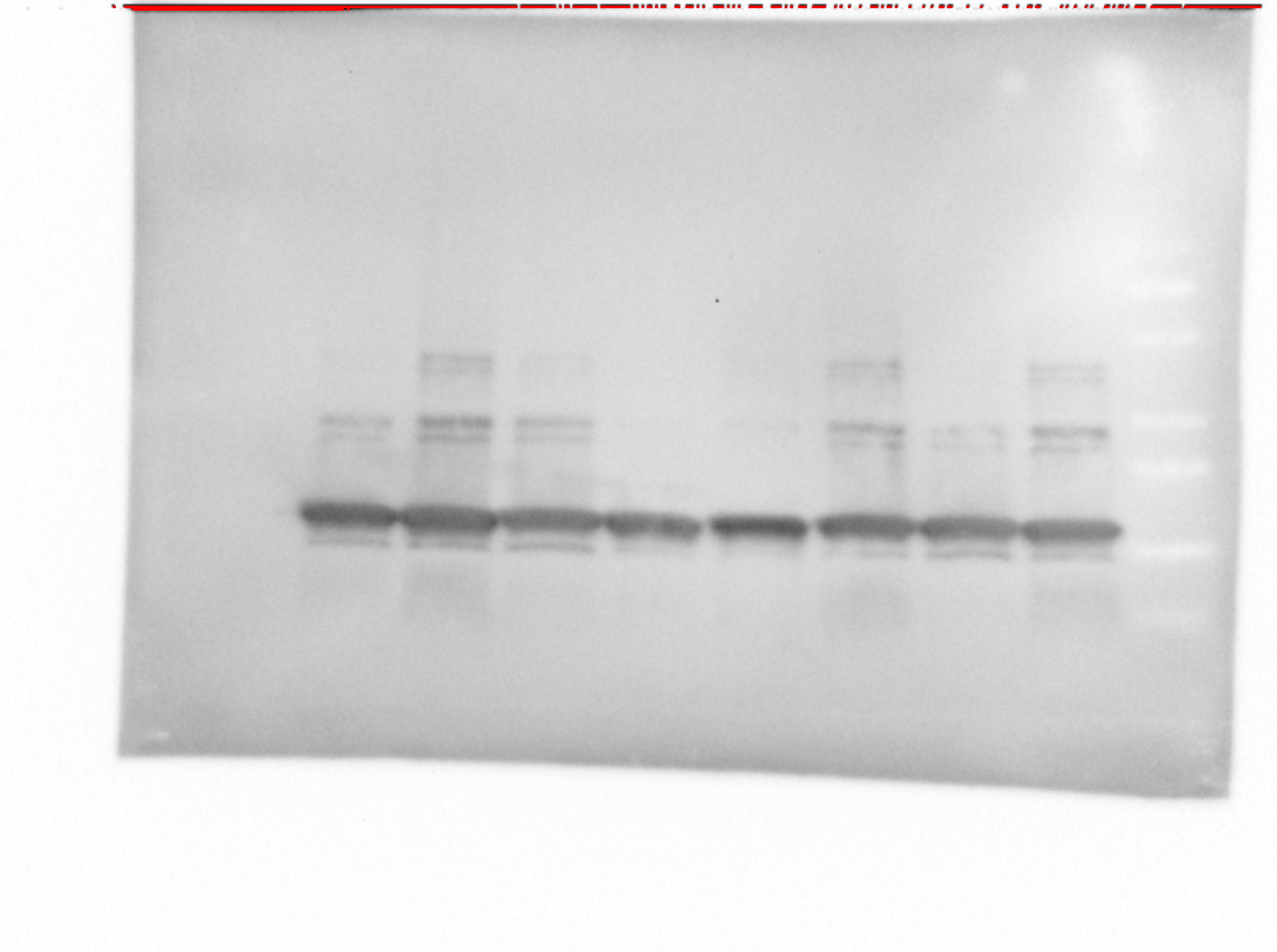

Supplement: Supplementary file 4 — Unprocessed western blots. [file 41551_2024_1190_MOESM4_ESM.tif]

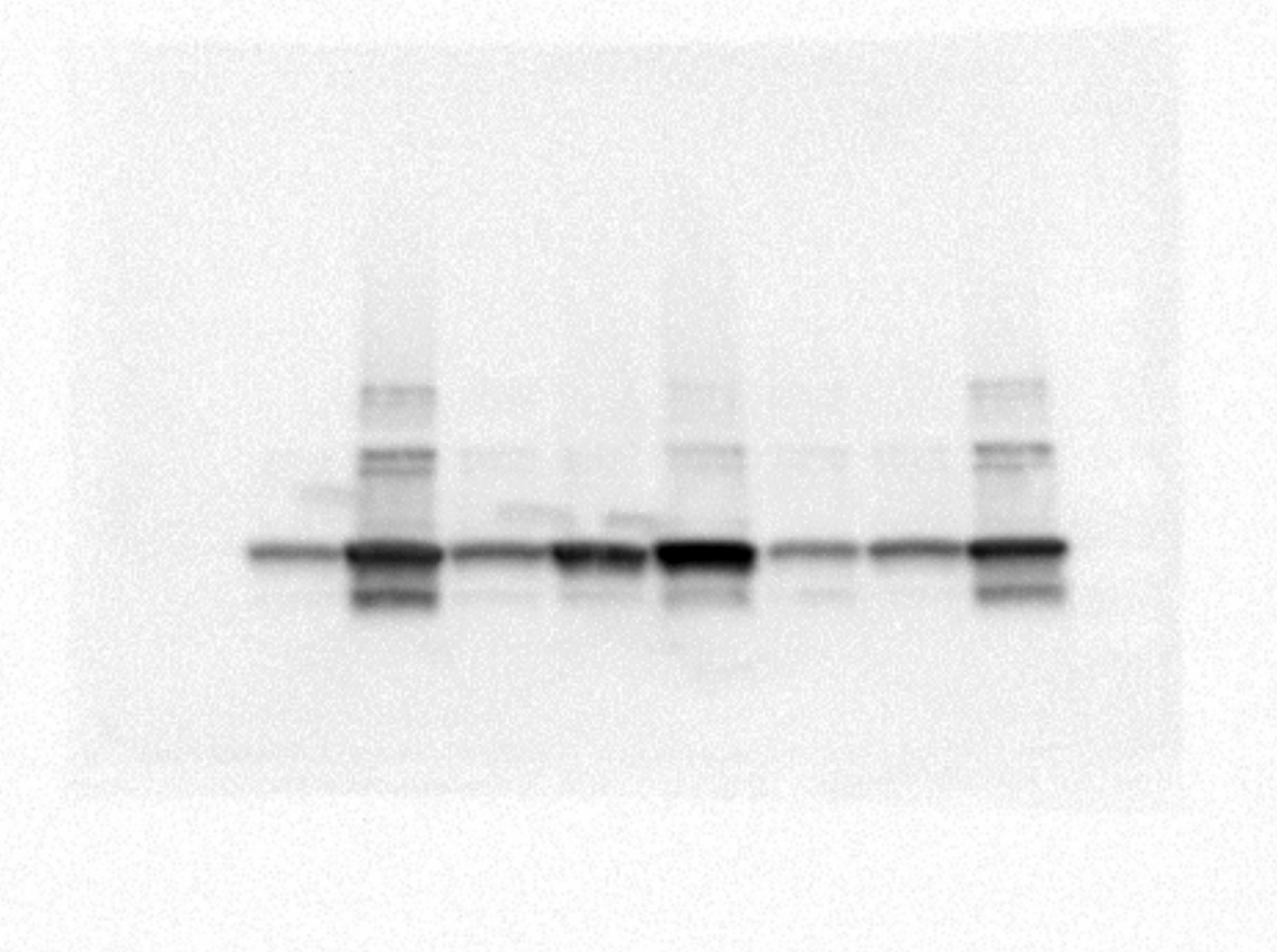

Supplement: Supplementary file 5 — Unprocessed western blots. [file 41551_2024_1190_MOESM5_ESM.tif]
